# Current Developments in Analytical Methods for Advanced Glycation End Products in Foods

**DOI:** 10.3390/molecules30204095

**Published:** 2025-10-15

**Authors:** Hiroyuki Kataoka

**Affiliations:** School of Pharmacy, Shujitsu University, Nishigawara, Okayama 703-8516, Japan; hkataoka@shujitsu.ac.jp

**Keywords:** advanced glycation end products (AGEs), glycation stress, Maillard reaction, sample preparation, analytical methods, liquid chromatography-tandem mass spectrometry, food

## Abstract

Advanced glycation end products (AGEs) derived from food are compounds readily formed during heating and processing through non-enzymatic glycation reactions such as the Maillard reaction. Since a variety and quantity of AGEs are formed within shorter times in food than in the body, their long-term excessive intake is a growing concern as a contributing factor to the onset of various diseases, including diabetes and age-related diseases. Therefore, investigating the formation and presence of AGEs in food and understanding their contribution to health risks has become critically important. Since AGEs with different characteristics exist in various forms in foods, it is essential to develop efficient sample preparation and sensitive and accurate analytical methods. Generally, analysis of free AGEs requires deproteinization, and bound AGEs are hydrolyzed using hydrochloric acid or enzymes to form free AGEs, which are then purified by defatting, reduction, and solid-phase extraction. While immunological techniques and instrumental analytical methods such as chromatography have been developed for the detection and analysis of AGEs, liquid chromatography-tandem mass spectrometry is widely used due to its high sensitivity, specificity, and operability. This review summarizes trends and challenges in sample preparation and analytical techniques for analyzing AGE formation and presence in food, based on papers reported over the past 20 years.

## 1. Introduction

In recent years, as quality of life has improved, awareness of high-quality food has grown significantly in relation to health and longevity. Consumers now pay close attention not only to food color, aroma, taste, and texture, but also to its nutritional value, quality, and safety. For example, heat-based cooking methods such as steaming, boiling, baking, and frying have become essential steps in modern food preparation. They not only enhance flavor and appearance to stimulate appetite but also extend shelf life and reduce the risk of food poisoning [1,2,3]. Furthermore, the diversification of diets, the development of various ingredients, and advances in food processing technology have led to a rapid increase in the consumption of highly heat-processed foods such as baked goods, canned foods, instant foods, and fast food [4]. However, heating food triggers the Maillard reaction, a complex interaction between free amino groups (derived from amino acids or proteins) and carbonyl groups (derived from sugars or aldehydes) [1,3,5], ultimately forming advanced glycation end products (AGEs) that can have adverse health effects [3]. In particular, processed foods, which are rich in sugars and proteins, can rapidly produce AGEs upon heating [2]. Consequently, with the widespread adoption of modern processed foods, excessive intake of AGEs is a growing concern [6,7,8].

AGEs are a type of compound produced by a series of irreversible non-enzymatic reactions between the amino groups of amino acids in proteins and the carbonyl groups of reducing sugars. They can be classified into two categories based on their origin: endogenous and exogenous [1,4,8,9]. Most endogenous AGEs are produced in vivo and accumulate in the body under physiological metabolic conditions, as well as during normal aging and metabolic diseases. Exogenous AGEs are primarily derived from dietary intake, but may also be derived from external environmental factors such as smoking and ultraviolet (UV) exposure. In the case of dietary sources, AGE formation via this glycation reaction occurs more rapidly during food processing involving heating, leading to the formation of large quantities of heterogeneous AGEs and their precursors within a relatively short period [8,10,11]. Furthermore, animal-based foods high in fat and protein content contain significantly more AGEs than plant-based foods rich in water, antioxidants, and vitamins. Particularly, high-fat and high-sugar browning foods that are cooked and processed at high temperatures contain large amounts of AGEs [3,8,12,13]. The level of AGEs in heat-treated foods can be 10 to 100 times higher than in untreated foods, making cooking and food processing critical factors determining AGEs content in food [14].

When these endogenous and exogenous AGEs accumulate in various tissues and organs, they bind to the receptor for AGEs, directly promoting the degradation of the extracellular matrix or activating intracellular signaling pathways, leading to cellular damage and the inhibition of normal physiological functions [1,2,4,8,13,15]. Recent observations suggest endogenous and exogenous AGEs may act synergistically, contributing to the overall glycotoxin burden and potentially increasing AGE-related health hazards [16]. Generally, total AGEs levels in the body are determined by a combination of dietary intake, genetics, age, and overall health status. Consequently, intake and accumulation of excessive AGEs, along with elevated systemic AGEs concentrations, can cause structural changes in proteins in the body, synergistically inducing excessive oxidative stress and inflammation in cells, potentially resulting in various health problems [8,15,16,17,18,19,20,21,22]. Previous studies have demonstrated that AGEs contribute to the progression of various chronic diseases [23], including diabetes [18,24] and its complications [11,25,26,27,28], chronic kidney disease [13,29], cardiovascular diseases such as atherosclerosis [13,19,30], cognitive disorders such as Alzheimer’s disease [31], neurodegenerative disorders [13,32,33], and aging-related diseases [19,34]. Furthermore, numerous studies have reported associations with metabolic disorders, bone degenerative diseases, and diseases of the lungs [35], liver [36], intestines [37], and reproductive system [13,18,38,39]. On the other hand, although the intestinal absorption rate of dietary AGEs (d-AGEs) is only 10–30% [18,40], they easily accumulate in the body via the bloodstream [8,19,41]. Therefore, the harmful effects of d-AGEs on the human body cannot be ignored [12], as long-term excessive AGE intake contributes to various physical dysfunctions that lead to the development of these diseases [2,8,12,42,43]. Indeed, d-AGEs are also attracting attention as a predictive risk factor for chronic diseases and related mortality [44]. However, among the various AGEs present in food, little is known about which AGE species have what level of toxicity.

Foods are the main source of AGEs in the human body, making controlling AGE intake crucial [45]. Previous studies have shown that food components (sugars, proteins, fats, water, additives, etc.), storage methods (temperature, time, humidity, etc.), processing methods (steaming, boiling, frying, baking, roasting, etc.), and processing conditions (pH, temperature, etc.) influence AGEs formation [3,8,12,45,46]. Furthermore, AGEs in food are categorized into free and bound forms, differing in bioavailability, digestion, absorption, excretion, interactions with gut microbiota, metabolites, and metabolic pathways [1,16,36,40,47]. Therefore, understanding their fate in the body after food ingestion is essential [8,40,48]. In addition, AGEs can potentially cause metabolic dysfunction in the body, so mitigating their harmful effects is important [8,9,49]. However, the lack of clear guidelines regarding d-AGE intake levels and their adverse effects on human health makes the establishment of explicit safety standards for consuming AGE-containing foods an urgent priority [8,10].

Currently, extensive research is being conducted on effects of food storage and processing on AGE formation, methods to suppress AGEs formation in foods, and ways to reduce AGEs intake from foods [8,9,11,49,50,51,52,53]. However, the detection and identification of AGEs in foods is extremely difficult due to the wide variety of AGEs and the complexity of food composition and the glycation reaction process. Furthermore, the lack of standardized analytical methods makes it difficult to compare data obtained between laboratories. Therefore, as heat-processed foods are expected to continue occupying a significant portion of the food industry, the development of effective analytical methods and characterization techniques for AGEs present in cooked and processed foods is essential for preventing AGEs-related health hazards and advancing AGEs research [3,8]. This review focuses on the formation and presence of AGEs in food and summarizes recent advances in sample preparation and analytical methods for their analysis, based on papers reported over the past 20 years.

## 2. Formation of Various Types of AGEs in Foods

### 2.1. Mechanism of AGE Formation

**Figure 1:**
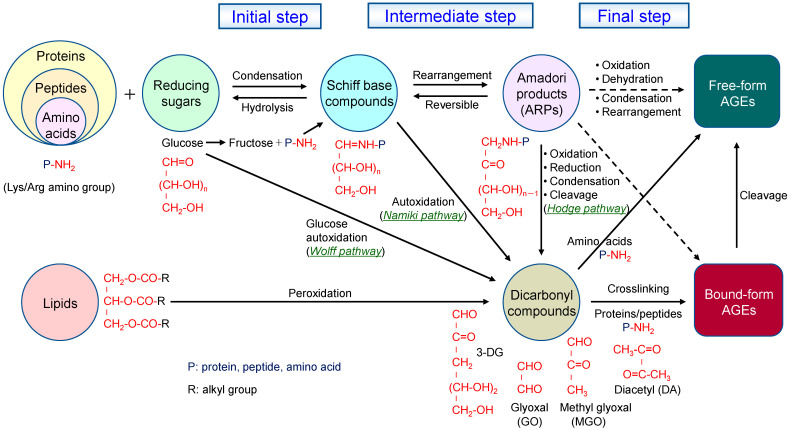
Possible pathways for the formation of AGEs.

In the Maillard reaction, reducing sugars (e.g., glucose, fructose, ribose) and their α-dicarbonyl compounds react with free amino groups (α-amino and ε-amino) and guanidino groups in proteins, peptides, and amino acids, followed by further rearrangements to form stable, irreversible end-products called AGEs [1,2,3,4,5,11,12,45,54]. Recent studies have also revealed that imidazole groups and indole are involved in the Maillard reaction, and that sulfhydryl groups react with carbonyl groups via a similar nucleophilic reaction [48]. The mechanism of AGE formation through these glycation reactions has been well analyzed in a Maillard reaction model system involving amino acids and reducing sugars [55,56,57,58,59,60]. In the Hodge pathway, which is the most important pathway, this non-enzymatic glycation process generally consists of three sequential steps: initial, intermediate, and final (Figure 1) [10,11,45]. In the initial step, the carbonyl groups of reducing sugars, such as aldoses and ketoses, react with primary amino groups (especially lysine (Lys) and arginine (Arg) residues) in proteins via a nonenzymatic aminocarbonyl reaction to form Schiff bases (imine compounds) [12,13,27,45]. This reaction is unstable and reversible, and either undergoes hydrolysis again or proceeds to an intermediate step where spontaneous intramolecular rearrangement leads to the relatively stable covalent Amadori rearrangement products (ARPs) [11,12,13,27,45,49,54,61]. This Amadori rearrangement reaction depends on pH and temperature. At pH ≤ 7, 1,2-enolization prevails, and ARPs decomposes into highly reactive α-dicarbonyl compounds such as 3-deoxyglucosone (3-DG), converting to furfural and methylfurfural. In contrast, at pH > 7 and low temperatures, 2,3-enolization process dominates or Strecker degradation reaction occurs, in which ARPs are transformed to dehydroreductones and reductones, subsequently leading to the generation of aldehydes and α-aminoketones. Furthermore, at pH > 7 and high temperatures, ARPs are converted to active 1,2-dicarbonyl compounds such as glyoxal (GO), methylglyoxal (MGO), and diacetyl (DA). These active dicarbonyl compounds can also be generated via the Wolff pathway (glucose autooxidation or amino-induced degradation), the Namiki pathway (oxidative cleavage of Schiff bases), and lipid peroxidation. In the final step, the ARPs irreversibly generate various AGEs through a series of complex reactions including dehydration, cyclization, fragmentation, oxidation, reduction, condensation, rearrangement, and isomerization [11,12,13,45,54]. Furthermore, the reactive dicarbonyl compounds formed at intermediate stages react with the amino groups of proteins (or peptides), causing cross-linking within and between proteins. Subsequently, they undergo oxidation, dehydration, or polymerization [11,12,18,61], resulting in the formation of stable protein/peptide-bound AGEs and free-form AGEs derived from amines or amino acids produced by their degradation [2,10,11,12,13,14,15,18,45]. In this way, the glycation reaction in food proceeds through various pathways, resulting in the formation of AGEs of various sizes [3]. However, food contains various matrix components, and many unknown AGEs that cannot be identified in simple model systems may be generated. Therefore, to control AGE formation in specific foods, it is considered necessary to construct and analyze formation models that simulate actual cooking and processing conditions.

### 2.2. Classification of Various AGEs

**Figure 2:**
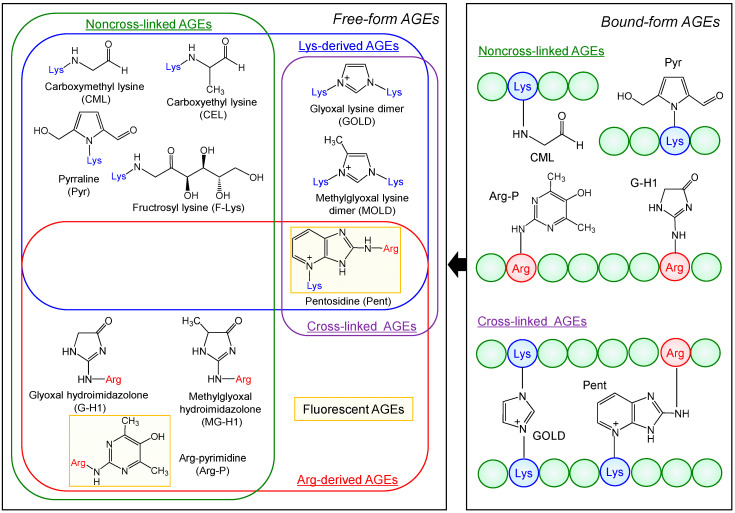
Structures of typical free-form and peptide/protein bound AGEs derived from Lys, Arg, or both in foods.

AGEs formed through the above series of complex reactions are classified based on molecular weight into bound AGEs containing high-molecular-weight peptides/proteins and free AGEs containing low-molecular-weight peptides (<5 kDa) or amino acids [8,16,48]. However, a clear boundary distinguishing the two has not been established [11,45]. Generally, AGEs often refer to free-type glycation products containing low-molecular-weight amino acids [31,62]. Currently, over 40 types of AGEs have been identified and characterized [2,3,4,11,40,63]. Figure 2 shows the chemical structures of various representative free AGEs and their corresponding bound-type AGEs. Free-type AGEs are primarily classified based on the type of amino acid reacting with the carbonyl group of the reducing sugar: Lys-derived, Arg-derived, and Lys/Arg-derived. Lysine-derived AGEs include *N*-ε-carboxymethyl lysine (CML), *N*-ε-carboxyethyl lysine (CEL), fructosyl lysine (F-Lys), pyrraline (Pyr), glyoxal lysine dimer (GOLD), methylglyoxal lysine dimer (MOLD), deoxyglucosoneimidazolone (DOLD), among others. Arg-derived AGEs include glyoxal hydroimidazolone (G-H1), methylglyoxal hydroimidazolone (MG-H1), Arg-pyrimidine (Arg-P), and Lys/Arg-derived AGEs such as pentosidine (Pent). AGEs are further classified into cross-linking and non-cross-linking AGEs based on their binding affinity to proteins. The Lys dimers GOLD, MOLD, and DOLD, as well as the Lys/Arg-derived Pent, are involved in forming cross-links within and between proteins (Figure 2). Furthermore, based on optical properties, AGEs are classified as fluorescent and non-fluorescent. In Figure 2, Arg-P and Pent exhibit fluorescence and are utilized for fluorescent detection of AGEs. Additionally, Pyr, GOLD, MOLD, G-H1, MG-H1, Pent, and Arg-P absorb ultraviolet light, enabling UV detection. However, CML, CEL, and F-Lys exhibit neither ultraviolet absorption nor fluorescence [1,4,40,45,48,64,65,66].

CML and CEL are formed by the reaction of Lys residues in proteins with GO and MGO, respectively, and are AGEs with excellent chemical and biological characteristics [3,13,45,66]. Pyr is a pyrrole derivative containing the *N*-ε-amino group of Lys and is formed during the reaction of Lys with 3-DG [40]. CML, CEL, and Pyr are well-studied markers for AGE formation in foods [45,65,66]. In addition to Lys-derived AGEs, the guanidino group of Arg functions as an alternative major reaction site in the Maillard reaction. Pent is a cross-linking AGE formed when Lys and Arg residues in proteins bond via a pentose linkage. Furthermore, the reactions of Arg with GO, MGO, and 3-DG yield hydroimidazolones derived from GO (G-H1), MGO (MG-Hs: MG-H1, MG-H2, and MG-H3), and 3-DG (3DG-H1), respectively [4]. Arg-P is formed by the reaction of Arg with two molecules of MGO [4], while GOLD, MOLD, and DOLD are formed by the reactions of two Lys side chains with GO, MGO, and 3-DG, respectively. Furthermore, similar cross-linking reactions between one Lys residue and one Arg residue generate GODIC (GO-derived imidazolium cross-link with Lys/Arg), MODIC (MGO-derived imidazolium cross-link with Lys/Arg), and DODIC (3-DG-derived imidazolium cross-link with Lys/Arg) [4,45]. However, no AGEs related to cross-linking between two Arg residues have yet been identified.

## 3. Analytical Methods for the Determination of AGEs in Food Samples

Since numerous AGEs with diverse structures exist in the complex matrix of food, appropriate analytical procedures are required to measure AGEs in food. Furthermore, as AGEs exist in food both as free forms and as peptide/protein-bound forms, they must be efficiently extracted and separated from the sample for accurate analysis. Generally, bound AGEs are measured as free AGEs after hydrolysis, and then detected and quantified by instrumental analysis or immunoassays [1,3,4,8,11,40,41,45,48,64,65,66]. The most commonly used instrumental analytical methods are high-performance liquid chromatography (HPLC) and liquid chromatography-tandem mass spectrometry (LC-MS/MS). Recently, ultra-high-pressure liquid chromatography (UHPLC)-MS/MS, enabling rapid analysis under high pressure and low flow rates, has also become available. Mass spectrometer-based methods provide highly sensitive and specific results, but require expensive equipment and complex and tedious sample preparation. On the other hand, the enzyme-linked immunosorbent assay (ELISA) is faster and simpler than instrumental analysis methods, but it suffers from issues such as lack of specificity and reproducibility, and interference with food matrices [40]. Therefore, while the ELISA method can be used for screening AGEs in food, it is difficult to accurately and simultaneously measure the various chemical species of AGEs present in food [40]. Over the past 20 years, various methods for measuring AGEs in different foods have been developed, and an overview of these analytical methods is summarized in Table 1. Most of these methods target CML or CEL, with few methods simultaneously analyzing other AGEs. This section introduces these methods, categorized by sample preparation and separation/detection techniques, citing application examples to verify their characteristics and usefulness.

### 3.1. Sample Preparation Techniques for Pretreatment and Extraction of AGEs in Food Samples

To accurately quantify AGEs with differing chemical and physical properties in food samples using instrumental analysis, AGEs must be separated and purified prior to measurement. However, most currently available extraction methods target CML and CEL, with limited research on other AGE types, and establishing an extraction method suitable for all types of AGEs is difficult due to compound-specific stability issues [45]. Generally, to efficiently extract free-form AGEs from food samples, appropriate pretreatment methods are necessary depending on the characteristics of the target AGEs and the state of the sample. In contrast, to analyze bound-form AGEs, it is necessary to establish hydrolysis conditions to release low-molecular-weight AGEs from peptides or proteins. Alternatively, the total amount of both free and bound AGEs can be determined without distinguishing between them. On the other hand, sample preparation for ELISA-based immunoassays is relatively simple, requiring only the removal of fats and proteins [40,46]. Food samples contain impurities such as proteins, fats, and sugars in addition to AGEs, and their forms vary from liquid to semi-solid to solid. Therefore, appropriate pretreatment steps like precipitation separation, extraction, and defatting are necessary. Particularly, deproteinization is essential for the analysis of free AGEs, while acid or enzymatic hydrolysis is essential for the analysis of bound AGEs [4,8,11,40,45,46]. For the food AGE analysis methods listed in Table 1, the sample preparation steps for analyzing free AGEs and bound/total AGEs, along with their characterization, are summarized in Table 2 and Table 3. The performance levels are evaluated based on the reports in each paper in terms of time cost, operability, recovery rate, economic cost, etc., with higher scores indicating better performance.

#### 3.1.1. Sample Preparation for Free AGE Analysis

Generally, free AGEs are extracted using solvents that freely mix with water, such as water, acetone, MeOH, acetonitrile, or acidic water, due to their high polarity and water solubility [4,45]. As shown in Table 2, proteins are first denatured and precipitated using strong acids such as trichloroacetic acid (TCA) [91,92,98,117,118,126,127], nonafluoropentanoic acid (NFPA) [85,104,120], sulfosalicylic acid [108], or organic solvents like MeOH, acetone, acetonitrile [78,94,95,96,121]. Subsequently, it is common to separate and concentrate AGEs from the supernatant by solid-phase extraction (SPE), or if the supernatant contains fat, the fat is removed with n-hexane prior to AGE separation and concentration by SPE. Li et al. [78] compared four methods for extracting and purifying free CML from soy sauce samples: (A) dilution with MeOH:water (1:9), (B) dilution with MeOH:water (1:9) after protein removal with 75% MeOH, (C) SPE using C18 with the MeOH:water (1:9) dilution solution, (D) SPE of the MeOH:water (1:9) dilution solution after protein removal with 75% MeOH. They reported the effectiveness of SPE, finding the purification efficiency to be in the order D > B > C > A, with method D being most effective. Currently, SPE cartridges include reverse-phase polymers such as C18, Oasis HLB, Strata-X-C, and Cleanert PEP-2; strong cation exchange polymers like HyperSep SCX; and hybrid-mode polymers combining reverse-phase and strong cation exchange, such as Oasis MCX and TELOS neo PCX. SPE offers various advantages, including low cost, applicability to diverse detection techniques, and simplicity of procedure. However, the use of SPE for analyte extraction has several drawbacks, such as requiring pre-filtration to remove solid particles from the sample solution and the long device or sorbent conditioning times.

Zhang et al. [104] extracted CML using SPE with Strata-X-C after deproteinizing ground almonds with NFPA. Sun et al. [91] used TCA instead of NFPA for deproteinization to prevent column degradation and extracted free CML and CEL from raw and heat-treated meat using SPE with Oasis MCX. In contrast, Hu et al. [95] extracted free CML and CEL from butter cookies by adding water to the ground sample, simultaneously defatting and deproteinizing with CHCl_3_/MeOH (2:1, *v*/*v*), then purifying the supernatant using SPE with Oasis MCX. Wang et al. [121] extracted free CML and CEL from stewed meat samples by deproteinizing with an 80% acetonitrile-water mixture, adding water to the supernatant, defatting the solution with n-hexane, and then purifying it using SPE with HyperSep SCX. Furthermore, Lu et al. [127] recently developed a novel method using solid-phase microextraction (SPME) instead of SPE. SPME is a pretreatment method that reduces organic solvent use and enables simple, efficient extraction and concentration. They fabricated SPME fibers coated with covalently bonded organic frameworks (COFs), a porous crystalline polymer, as the adsorbent and applied them to the analysis of CML, CEL, and Pyr in heat-treated chicken and fish. After protein removal with TCA and defatting with n-hexane, the resulting solution was extracted and concentrated using SPME, followed by high-sensitivity analysis via UHPLC-MS/MS. This method achieves sensitivity more than 10 times higher than conventional methods using SPE and reduces solvent consumption.

As shown in Table 2, there are few reports on pretreatment methods for analyzing free AGEs in food. Generally, fewer pretreatment steps allow for simpler and faster processing, but insufficient removal of contaminants may compromise sensitivity and specificity. In contrast, increasing pretreatment steps improves separation and purification, but may reduce analyte recovery during the process. Considering these points, ensuring complete protein removal and avoiding adsorption of analytes to precipitates is crucial, and efficient extraction methods such as SPE or SPME an effective approach.

#### 3.1.2. Sample Preparation for Bound and Total AGE Analysis

Bound AGEs must be converted to free AGEs by hydrolyzing peptides or proteins using acid or enzymes [1,4,8,11,40,45,46,48,65]. Acid hydrolysis is often used for AGE analysis in foods due to its simplicity, cost-effectiveness, and reliability. However, some AGEs are unstable under hydrolytic processing. For example, during acid hydrolysis, the pyrrole ring of Pyr breaks down, and F-Lys also decomposes, converting to CML. Therefore, before hydrolyzing protein precipitate fractions, the sample must be reduced using a reducing agent such as sodium borohydride (NaBH_4_). Consequently, enzymatic hydrolysis is more suitable when targeting these acid-unstable AGEs. Enzymatic hydrolysis is commonly used for the analysis of hydroimidazolone isomers such as G-H1 and MG-H1, in addition to Pyr and F-Lys [4,8,45,48]. Furthermore, in the analysis of CML in milk samples using both acid hydrolysis and enzymatic hydrolysis, the protein hydrolysis rate was 94.3% for acid hydrolysis versus 86.3% for enzymatic hydrolysis [77], and a good correlation was observed between the two hydrolysis methods [103]. However, while these acid- or enzyme-based hydrolysis methods can be applied directly to food samples or after protein extraction, some small peptides or proteins may be lost during extraction from the food matrix. Therefore, for effective analysis of specific AGEs, the completeness of hydrolysis and recovery rate from the sample are critical, necessitating specialized sample preparation protocols [4,8,45,48,66]. As shown in Table 3, when analyzing bound AGEs, operations such as dialysis, reduction, defatting, and protein precipitation are required before acid or enzyme hydrolysis. Various methods are used depending on the sequence of these operations. Although hydrolyzed AGEs may sometimes be analyzed directly, an additional step of extracting and concentrating the released AGEs via SPE or SPME [127] is often performed to remove impurities. Furthermore, derivatization [67,68,69,72,73,84,114] may be applied to enhance compound stability and detection sensitivity.

Generally, reduction treatment with NaBH_4_ in sodium borate buffer (pH 9.2) is performed to convert complex chemical structures into more stable, simpler structures, thereby facilitating the extraction of AGEs. The temperature and time of the reduction reaction vary depending on the sample, ranging from 6–12 h or overnight at 4 °C, 4 h at 20 °C, or 1–4 h or overnight at room temperature. Because the presence of lipids may cause the formation of CML or CEL during acid hydrolysis [129], fat removal is necessary. For defatting, n-hexane, CHCl_3_:MeOH (2:1, *v*/*v*), or CHCl_3_:acetone (1:3, *v*/*v*) are commonly used. Additionally, TCA is often used as a protein precipitant due to its ease of removal after treatment. Acid hydrolysis typically involves a 24 h reaction at 110 °C in 6 M HCl, though reaction times are sometimes shortened to 18–23 h depending on the sample. Microwave-assisted acid hydrolysis involves a 1 min reaction at 150 °C in 6 M HCl followed by a 10 min reaction at 165 °C, significantly reducing hydrolysis time [71,75,130,132]. On the other hand, for enzymatic hydrolysis, a robust and reliable protocol is employed that involves sequential treatment using multiple hydrolytic enzymes with different specificities, such as pepsin, pronase E, prolidase, and aminopeptidase M, to enable its application to a wide range of food samples [1,4,45,48,65]. Furthermore, due to extended incubation times, antimicrobial compounds (e.g., thymol) may be added to the enzymatic reaction mixture [65], or for specific proteins, additional appropriate protease treatment may be introduced. Gómez-Ojeda et al. [83] digested samples using protease K, a subtilisin-related enzyme with broad cleavage specificity, to measure CML content in selected foods. Following acid hydrolysis and enzymatic hydrolysis, SPE is sometimes employed to separate AGEs from impurities in the hydrolysate. While acid hydrolysis requires first reducing F-Lys with NaBH_4_ to prevent its conversion to CML, enzymatic hydrolysis does not require this prior reduction [45]. For the purification of AGEs by SPE, cartridge columns with various stationary phases are used, as described in the sample preparation for free-form AGEs above. Among these, Oasis MCX, a mixed-mode polymer combining reverse-phase and strong cation exchange, is widely used. Conversely, for gas chromatography (GC)-MS analysis, conversion to volatile derivatives is necessary. CML is analyzed as trifluoroacetylmethyl ester derivatives via methylation with thionyl chloride/MeOH solution and acylation with anhydrous trifluoroacetic acid (TFA) [67,68,69]. Furthermore, for LC-MS analysis, derivatives of the amino group using o-phthalaldehyde (OPA), ethyl chloroformate (ECF), dansyl chloride (DNS-Cl), 9-fluorenylmethyl chloroformate (FMOC-Cl), etc., are also employed [72,73,84,114].

Wu et al. [90] analyzed protein-bound CML and CEL in egg white and yolk by reducing them with NaBH_4_ at 4 °C for 8 h, followed by simultaneous defatting and protein precipitation using CHCl_3_:MeOH (2:1, *v*/*v*) to simultaneously remove fat and recover protein precipitates. The resulting solution was hydrolyzed with 6 M HCl at 110 °C for 24 h and purified using SPE with Oasis MCX. Meanwhile, Chen et al. [112] reduced milk samples with NaBH_4_ at 4 °C overnight, hydrolyzed the solution with 6 M HCl at 110 °C for 24 h, and purified it using an SPE cartridge. This method eliminated the protein precipitation step. Comparing four SPE cartridges revealed that Oasis MCX most efficiently extracted CML. Jiao et al. [97] analyzed bound CML and CEL in 99 tea samples by defatting them with n-hexane, reducing them with NaBH_4_ at 4 °C overnight, hydrolyzing them with 6 M HCl at 110 °C for 24 h, and purifying the solution using SPE with Oasis MCX. Furthermore, Zhang et al. [26] developed extraction protocols for 334 different food items to construct a comprehensive database of nine types of AGEs in foods. Food samples were freeze-dried, degreased with CHCl_3_:MeOH (2:1, *v*/*v*) and simultaneously precipitated proteins. The samples were then reduced with NaBH_4_ at 25 °C for 3 h, followed by hydrolysis with 6 M HCl at 110 °C for 24 h. Meanwhile, Poojary et al. [132] developed a method for roasted meat and UHT milk involving simultaneous defatting and protein precipitation with CH_2_Cl_2_:MeOH (8:2, *v*/*v*), followed by microwave-assisted acid hydrolysis, successfully purifying 15 types of AGEs. Furthermore, Lee et al. [76] recently developed a novel method using precolumn derivatization based on enzymatic hydrolysis and dansylation to overcome the difficulties in chromatographic separation caused by the high hydrophilicity, diversity, and structural similarity of AGEs, and applied it to the analysis of 14 types of AGEs. After defatting freeze-dried powders of coffee, beer, and sausage with n-hexane, four proteases were reacted at 37 °C for three incubation times (24 h with pepsin, 24 h with pronase E, and 24 h each with leucine aminopeptidase and prolidase), and then the reaction mixture was derivatized with DNS-Cl. This method does not require purification via SPE and achieves improved sensitivity through derivatization.

On the other hand, a solution obtained directly by hydrolysis without protein precipitation or separation of free and bound forms is used for total AGE analysis [45]. In acid hydrolysis, samples are typically defatted with n-hexane, reduced with NaBH_4_, then hydrolyzed with 6 M HCl at 110 °C for 24 h. Free AGEs and AGEs released from bound forms are further purified by SPE. In enzymatic hydrolysis, samples are typically defatted with n-hexane, undergo multi-enzyme hydrolysis, and are further purified by SPE. Tareke et al. [85] measured the total CML and CEL content in porridge samples by directly hydrolyzing them with 6 M HCl at 110 °C for 24 h and then purifying them by SPE using TELOS neo PCX. Furthermore, Hegele et al. [103] determined total AGEs content in milk samples by fully hydrolyzing with a multi-enzyme system, then purifying CML, CEL, and F-Lys via SPE using Oasis HLB. On the other hand, knowing the amounts of both free and bound AGEs allows their summation to yield total AGEs. Liang et al. [84] measured the content of free Pyr from a protein-hydrolyzed beverage by SPE using Cleanert PEP-2, and measured the content of peptide-bound Pyr by SPE after enzymatic hydrolysis, calculating their sum as total AGEs. Therefore, if the total AGEs content is known, measuring either the free or bound AGEs content allows the respective bound or free AGEs content to be determined by subtracting it from the total AGEs content [48].

As shown in Table 3, although various methods have been reported for the pretreatment of bound and total AGEs in food, enzymatic hydrolysis is suitable for measuring AGEs unstable to acid hydrolysis, such as Pyr. However, enzymatic hydrolysis requires the use of multiple enzymes in a three-step reaction sequence to completely hydrolyze various proteins, necessitating a reaction time of at least three days. In contrast, acid hydrolysis can be completed within one day. Specifically, microwave-assisted acid hydrolysis enables decomposition within 10 min. However, with any acid hydrolysis method, caution is required not only regarding the degradation of some AGEs but also concerning the generation of new AGEs due to strong acidity and high-temperature heating. Additionally, protein fractionation, reduction treatment, and defatting operations must be applied according to the form of the food sample. Furthermore, SPE and SPME enhance purification efficiency, while derivatization methods effectively improve detection sensitivity and selectivity. Considering overall factors such as simplicity, speed, and cost of sample pretreatment operations, microwave-assisted acid hydrolysis is applicable to the analysis of many AGEs in foods and can be considered the most effective pretreatment method currently available.

### 3.2. Instrumental Techniques for Separation and Detection of AGEs in Food Samples

Methods for detecting AGEs in food are generally classified into two types: instrumental analysis and immunoassays. Instrumental analysis methods applicable to these include fluorescence spectroscopy, GC-MS, HPLC-UV detection, HPLC-diodes array detection (DAD), HPLC-FLD, LC-MS, LC-MS/MS, ultra-high-pressure liquid chromatography (UHPLC)-MS/MS, and ultra-performance liquid chromatography (UPLC)-MS/MS [1,3,8,11,40,45,48,65]. UPLC is the UHPLC instrument brand of Waters Corporation. However, fluorescence spectroscopy can only measure certain AGEs with fluorescent properties, such as Pent and Arg-P, and has drawbacks including the impact of fluorescence contamination from solvents or coexisting substances in the sample on accuracy [45]. GC-MS analysis requires derivatization to enhance volatility, because AGEs are highly polar and water-soluble. Therefore, methods combining HPLC or UHPLC separation with multiple detectors, such as UV, DAD, FLD, MS, and MS/MS, are widely used. However, derivatization is required for UV, DAD, or FLD detection of CML, CEL, and F-Lys, which do not exhibit UV absorption or fluorescence characteristics. Among these LC detectors, MS and MS/MS can selectively identify compound structures, do not require derivatization, and can be used for the detection of a wide range of AGEs. Specifically, the MS/MS method based on Multiple Reaction Monitoring (MRM), where selected parent ions fragment into specific daughter ions, enables even higher selectivity and more sensitive detection than MS alone. However, quantitative analysis typically requires the addition of stable isotope-labeled internal standards to correct for losses during sample pretreatment and matrix effects. Currently, the most commonly used instrumental analytical method is LC-MS/MS, which is more suitable for accurate quantification compared to other detectors for HPLC. Furthermore, using UHPLC allows for withstanding high pressures of 103–124 MPa and achieving good separation even at low flow rates. On the other hand, ELISA-based immunoassays are widely used because they do not require large equipment and are relatively simple to operate. They can be applied to both fluorescent and non-fluorescent AGEs, but they require specific antibodies against the target AGEs and have issues such as cross-reactivity [1,3,8,11,40,45,48]. Table 4 summarizes the advantages and disadvantages of instrumental analysis and immunoassays. The following outlines the main characteristics and application examples of key analytical techniques.

#### 3.2.1. GC-MS Methods

Although GC-MS is a selective and highly sensitive method, it cannot be applied to non-volatile or thermally unstable compounds. Conversion to stable volatile derivatives is essential, leading to few recent reports analyzing AGEs in food using GC-MS. Typically, GC-MS analysis of AGEs in food requires laborious and time-consuming sample pretreatment steps such as defatting, reduction, hydrolysis, and derivatization [3]. Derivatization is typically achieved by esterifying the carboxyl group with MeOH under strongly acidic conditions, followed by acylation of the amino group with trifluoroacetic anhydride. However, the derivatization process prior to measurement is relatively time-consuming [66,67,68,69]. Jiang et al. [68] analyzed CML in fried fish nuggets as trifluoroacetylmethyl ester derivatives using a DB-5 capillary column for GC-MS analysis. Similarly, Yaacoub et al. [69] developed an enhanced selective GC-MS/MS method for CML in roasted nuts and sesame seeds using the same derivative. GC-MS has also been applied to quantify CML in various food samples, including infant formula, milk, and meat [1,3,4,40,45,66]. However, CML contents in high fat samples are often too low to be detected by GC-MS [66]. Furthermore, since the precision and stability of GC-MS are lower than those of LC-MS, resulting in fewer applications for AGEs other than CML [3,85].

#### 3.2.2. HPLC and UHPLC Methods

HPLC is routinely used for the separation, identification, and quantification of components from complex matrices, and analytes separated by the column can be analyzed using multiple detectors combined with HPLC, such as UV, DAD, FLD, and MS [1,3,4,8,11,40,45,65,66]. On the other hand, UHPLC and UPLC, which are highly efficient and sensitive analytical techniques with improved resolution, require less solvent and have significantly reduced analytical cycle times due to their high-speed analysis, making them environmentally friendly and cost-effective alternatives to HPLC. However, they have drawbacks, such as high maintenance costs, due to the use of small columns operating under high pressure [8,11]. Detectors such as UV and DAD can detect substances with ultraviolet absorption capabilities, making them particularly suitable for measuring Pyr content in foods. Zhu et al. [70] used HPLC separation with a Sphere Clonek column and UV detection to measure free Pyr released from pectin oligosaccharides after enzymatic hydrolysis in infant formula milk powder, though the sensitivity was not particularly high. Similarly, Poojary et al. [71] developed a method for quantifying bound Pyr in ultrahigh temperature (UHT) milk using UPLC-DAD with an ACQUITY HSS T3 column after enzymatic hydrolysis. This method offers relatively high sensitivity, low noise, a wide linear range, and excellent selectivity. On the other hand, the HPLC-FLD method is commonly used for detecting fluorescent and cross-linked AGEs such as Pent and Arg-P [45]. This method exhibits high selectivity, high sensitivity, and good reproducibility. However, since it reacts only with fluorescent AGEs, non-fluorescent AGEs like CML require derivatization with a fluorescent compound. Derivatization can be performed using various reagents, such as OPA/2-mercaptoethanol [3,8]. Chen and Smith [72] and Öztürk et al. [73] measured CML contents in cooked chicken, pork, beef, and seafood using a pre-column derivatization method with OPA. After defatting with chloroform/MeOH (2:1, *v*/*v*), samples were reduced and acid-hydrolyzed. The resulting free CML was analyzed by HPLC-FLD using an OPA derivative and a reverse-phase TSK gel ODS-80 TM column. MS detection, on the other hand, offers high selectivity by detecting compound-specific ions, allowing for highly sensitive analysis of AGEs without derivatization; however, compounds with the same mass-to-charge ratio cannot be distinguished by MS alone and must be completely separated on the LC column. Zhang et al. [75] separated and quantified 14 types of AGEs derived from Lys, Arg, and cysteine in UHT milk using LC-MS with a reverse-phase Peptide XB-C18 column. The supernatant deproteinized with MeOH was designated as the free AGEs fraction, while the microwave-assisted acid hydrolysate served as the total AGEs fraction. In this method, Pyr was quantified after enzymatic hydrolysis instead of acid hydrolysis. Furthermore, Lee et al. [76] developed a method for the simultaneous, high-precision, and high-sensitivity analysis of 14 AGEs as DNS derivatives using UPLC-DAD-MS, applying it to the analysis of foods such as coffee, beer, and sausage, as well as glycated proteins. After degreasing the sample, enzymatically hydrolyzed free AGEs were derivatized with DNS-Cl and mutually separated within 16 min on a BEH C18 column, achieving detection sensitivity at the ng/mL level. This novel pre-column derivatization method overcame the difficulty of quantification caused by the high hydrophilicity and structural diversity of AGEs [89,90,91,92,93].

#### 3.2.3. LC-MS/MS Methods

LC-MS/MS is more sensitive than UV or FLD detection, does not require derivatization, and complete separation on the LC column is not necessary if detected in MRM mode. It can identify and quantify target analytes with high accuracy and resolution, and is the most widely used method due to its ease of operation [3,8,40]. However, compound recovery rates during sample preparation can be low, and the presence of co-extractants in the sample matrix can affect the ionization efficiency of the electrospray ionization (ESI) source, potentially causing ion enhancement or ion suppression of the target analyte [46]. Therefore, standard addition and isotopic internal standard dilution methods are usually used to correct for strong matrix interference peaks and ion suppression effects arising during the solid-phase extraction and ionization processes [3,46]. Furthermore, since AGEs are highly polar compounds, analysis using reverse-phase columns (e.g., C18) cannot completely separate individual AGEs. Consequently, ion-pair chromatography has been employed to enhance column separation [132]. However, prolonged use of ion-pair reagents may degrade ESI interface performance and reduce signal intensity for the analyte [128]. To avoid these issues, hydrophilic interaction liquid chromatography (HILIC) has been used to separate AGEs [89,90,91,92,93]. As listed in Table 1, LC-MS/MS methods have been applied to analyze various foods, enabling simultaneous analysis of multiple types of AGEs. The method using UHPLC-Orbitrap-Q-Exactive-MS has achieved the lowest detection limits. However, these methods require sample pretreatment to maximize the removal of impurities that can affect analytical accuracy [46]. Compared to LC-MS/MS, UPLC-MS/MS offers superior performance in terms of selectivity, recovery rate, repeatability, and reproducibility, while significantly reducing solvent consumption and analysis time. However, it has drawbacks such as high costs for acquiring and maintaining the equipment [3,11,45]. Eggen and Glomb [109] developed a highly sensitive method for analyzing 11 types of AGEs derived from GO and MGO generated during grilling pork using QTrap LC-ESI-MS/MS with an HSS T3 column. Lin et al. [128] developed a highly sensitive analytical method for 11 types of AGEs derived from α-dicarbonyl, Lys, and Arg in commercially available canned meat and seafood products using UHPLC-ESI-MS/MS with a BEH amide column. Meanwhile, Poojary et al. [132] proposed a method for analyzing major AGEs in processed foods, using an Orbitrap mass spectrometer (UHPLC-MS/MS) with a reverse-phase C18 column. They separated and detected 15 types of AGEs in samples, enabling the analysis of AGEs and protein cross-linking in complex matrices. However, this method has the problem of low recovery rate despite its high sensitivity. Furthermore, Zhang et al. [131] developed a highly sensitive, specific, and rapid UHPLC-QqQ-MS/MS method to quantify nine major AGEs. They constructed a comprehensive AGE database for 334 food items, enabling accurate assessment of dietary exposure to these glycotoxins and investigation of their physiological effects on human health. Additionally, LC-MS/MS methods are utilized in various fields, including analyzing AGE formation mechanisms and in vivo dynamics, controlling formation via cooking conditions, and monitoring milk processing [1,3,4,8,11,40,45,48,65,66].

#### 3.2.4. ELISA Methods

ELISA methods enable rapid and simplified sample testing processes. While AGE analysis kits are widely used and popular in the medical field, no kits specifically designed for food analysis exist; instead, clinical analysis kits are repurposed [1,3,4,11,40,45,66]. Although some AGEs share common antigenic determinants, allowing polyclonal and monoclonal anti-AGE antibodies to react with both free and bound AGEs, many studies have demonstrated cross-reactivity [1,3,45,66]. However, Amadori products (early products of the Maillard reaction) do not react with these antibodies, allowing ELISA to specifically detect AGEs in samples. Furthermore, while ELISA methods are useful for establishing preliminary databases of AGE content in various foods, analytical results tend to be significantly biased because the food matrix greatly influences the specific antigen–antibody binding reaction [40]. Indeed, when using the ELISA method, it is important to note that AGEs levels in high-fat foods such as cheese, dairy products, butter, and olive oil may be overestimated, while AGEs levels in starchy foods may be underestimated [3,45,83]. Therefore, to verify the practical validity of the ELISA method, proper antibody characterization (including target epitope, cross-reactive structures, and matrix effects) is necessary [4]. Furthermore, implementing ELISA as a detection method for food samples presents several drawbacks, such as requiring trained personnel and specialized equipment [11]. However, recent analyses have utilized commercially available ELISA kits for clinical use, which are relatively simple to operate. Prosser et al. [134] measured CML in hydrolyzed milk whey protein using a commercially available CML ELISA kit from Echelon Biosciences in a relatively short time. They also identified which proteins in milk and powdered milk samples were CML-modified using immunoblotting with anti-CML antibodies. Meanwhile, Altun et al. [135] digested cooked meat samples with a protease K solution and then measured both CML and CEL in the samples using separate OxiSelectTM competitive ELISA kits. Although both ELISA kits require a 24 h incubation period, the procedures are relatively simple.

#### 3.2.5. Comparison of Various AGE Analysis Methods

Both LC-MS/MS and ELISA have been widely used to quantify AGEs in foods. However, direct comparison between the two methods is difficult because LC-MS/MS results are expressed as concentrations (e.g., mg kg^−1^ protein or mg kg^−1^ food), whereas ELISA results are reported as arbitrary units (e.g., kilo units per gram of food) rather than actual concentrations [45]. Furthermore, ELISA targets the entire Maillard reaction product, making it less specific for particular AGEs, further complicating comparison with LC-MS/MS. Gómez-Ojeda et al. [83] compared three different ELISA methods with LC-MS/MS and found no correlation between the ELISA kit and LC-MS. Charissou et al. [67] compared ELISA and GC-MS for the measurement of CML in dairy products, finding ELISA yielded values 10 times higher. Additionally, several studies reported CML levels in Cheddar cheese measured by ELISA being approximately 73-fold higher than those measured by UPLC-MS/MS [66]. One reason for this significant discrepancy may be the use of two different cheese brands, but it may also be due to interactions between the food matrix and the anti-CML antibodies in ELISA. Therefore, the ELISA method only provides semi-quantitative results and is suitable for screening multiple samples of products with relatively high AGEs concentrations. For more accurate measurements, instrumental analysis is necessary. Indeed, Tareke et al. [85] measured CML in porridge samples using GC-MS, LC-MS/MS, and ELISA methods, demonstrating that LC-MS/MS outperformed the other two methods in terms of reproducibility and specificity.

As shown in Table 4, the most commonly used instrumental analytical methods currently are LC-MS/MS and UHPLC-MS/MS. Although these instruments are expensive, they offer superior specificity, sensitivity, and accuracy compared to other methods and can analyze multiple types of AGEs. However, the detection limits (LOD) for CML by LC-MS/MS in Table 1 vary widely, ranging from several ng to several μg. This may be due to differences in the performance of the measuring instruments or separation columns. Furthermore, the units used to express the measured values differ across papers—some are expressed in ng/mL, while others use ng/g sample, μg/g protein, mmol/mol Leu, etc.—making comparison of the respective data difficult. Furthermore, as shown in Table 1, Table 2 and Table 3, the diverse sample preparation methods used prior to introducing samples into these instruments affect the sensitivity and accuracy of the instrumental analysis. This ultimately leads to variations in reproducibility and recovery rates across different measurement institutions. Therefore, standardizing sample preparation methods and analytical procedures, considering simplicity and cost, is a future challenge.

## 4. Presence and Contents of Main AGEs in Various Commonly Consumed Foods

Since d-AGEs contribute to the AGE pool in the body [4,43], it is important to understand the AGE content in foods and the intake of d-AGEs when evaluating the health effects of AGEs. To date, various analytical methods have measured the presence and content of AGEs, and it has been reported that numerous AGEs with different structures exist in various foods [109,110,131,132]. Table 5 summarizes the content of major AGEs in various foods, categorized by food type and divided into free and bound AGEs. It shows that diverse AGEs are widely present in foods, and their content is closely related to raw materials and cooking/processing methods. In particular, animal-based foods rich in fat and protein typically contain abundant AGEs, and cooking processes such as frying, grilling, and baking tend to further increase AGEs. In contrast, plant-based foods like fruits and vegetables, which are high in water, antioxidants, and vitamins, generally have lower AGEs content and show minimal increases even after cooking [85]. Furthermore, AGEs formation is promoted not only by heat cooking but also by common food processing methods such as roasting and heat sterilization [91,92,104]. Generally, the AGEs levels in heat-treated foods are 10 to 100 times higher than in untreated foods [14]. Therefore, not only the type of food but also the cooking, processing, and storage methods are important factors determining d-AGEs intake.

In modern diets, we tend to consume large amounts of AGEs from cooked fish, poultry, and meat; powdered milk and cow’s milk; and processed grain products like cereals and bakery items. Daily AGEs intake, calculated from CML and Pyr content, is estimated to be 25–75 mg day^−1^ [4,16]. However, this is based on only a portion of the AGE content, and it is also necessary to understand the types of AGEs and their contribution to toxicity. From previous analytical examples, CML, CEL, and MG-H1 have been detected in relatively high concentrations in foods, but reports covering multiple types of AGEs are limited. Regarding the form of AGEs, it has been reported that soy sauce and beer contain significantly higher levels of free AGEs than bound AGEs [107,108]. In contrast, bound AGEs are reported to be relatively abundant in meat and meat products, milk and dairy products, nuts, and protein hydrolysate-enriched beverages [91,92,104]. However, few reports clearly distinguish and measure the content of free AGEs and bound AGEs in foods; measurements are often reported as total AGEs. Recently, a comprehensive AGE database covering 334 food items and 9 major AGEs was constructed. This enables accurate assessment of dietary exposure to these glycotoxins and investigation of their physiological effects on human health [131]. However, there is no reliable data on the acceptable daily intake of AGEs for healthy individuals.

### 4.1. Meat and Meat Products

As shown in Table 5, most detected AGEs in meat and meat products are CML and CEL. Recently, with the development of new analytical methods, compounds like Pyr [127] and MG-H1 [123,125] are also being measured simultaneously. Furthermore, Lin et al. [128] simultaneously measured Lys-derived AGEs (CML, CEL, GOLD, MOLD, etc.) and Arg-derived AGEs (G-H1, MG-H1, Arg-P, etc.) in commercially available canned meat and seafood products using UHPLC-MS/MS. Canned foods generally undergo heat sterilization to ensure long-term shelf life; however, these stringent heat treatment conditions can potentially lead to AGE formation. CML, CEL, G-H1, and MG-H1 were detected in all canned meat and seafood products across a total of 10 groups, with CEL and MG-H1 being particularly abundant. MOLD and GOLD were relatively abundant in canned pork but were scarcely detected in canned sardines, clams, or tuna. Conversely, Arg-P content was relatively low and was not detected in any canned tuna, sardine, or clam samples. The total AGEs content in canned foods was high in canned pork (318 μg g^−1^), snails (330 μg g^−1^), Pacific saury (349 μg g^−1^), mackerel (308 μg g^−1^), and eel (326 μg g^−1^), while it was relatively low in canned Spam (139 μg g^−1^), chicken (155 μg g^−1^), tuna (112 μg g^−1^), and clams (112 μg g^−1^). Among AGEs, CML and CEL are particularly abundant [3], with bound forms being more prevalent than free forms in raw fish, chicken, pork, and beef, but this differed between animal strains, between individual muscles within an animal, and between meat from different suppliers [91]. Furthermore, AGE content in meat products is primarily related to heat treatment processes, meat type, and food additives. Cooking or heating methods were also shown to have various effects on the levels of both free and protein-bound AGEs in meat, poultry, and fish products. For example, heating (100 °C, 5 min, 10 min, 30 min) or heat sterilization (121 °C, 10 min) significantly increased the protein-bound CML and CEL content in fish muscle, pork, beef, and chicken, but had little effect on the free CML and CEL content [8,40,85,86]. Furthermore, increases in CML and CEL content have been observed under cooking conditions such as microwave heating, addition of reducing sugars, and salt addition [85,109].

### 4.2. Eggs and Dairy Products

As shown in Table 5, reports on chicken eggs are limited. Wu et al. [90] measured protein-bound CML and CEL in raw and heat-treated egg whites and yolks using LC-MS/MS. Significant variation in these AGE contents was observed between individual raw eggs. Yolk contained higher levels than egg white, attributed to its higher protein content and greater susceptibility to lipid oxidation. Heating significantly increased CML content in both egg white and yolk, while decreasing CEL content in yolk. Zhang et al. [131] found that besides CML and CEL, G-H1, MG-H1/3, MG-H2, GOLD, and MOLD were also present in boiled and marinated eggs at low to moderate levels, and reported that stir-frying significantly increased AGE levels.

Meanwhile, numerous reports exist on the presence and content of AGEs in milk and dairy products [66] (Table 5). AGEs in these foods are primarily CML, CEL, F-Lys, Pyr, G-Hs, MG-Hs, GOLD, and MOLD, with their levels varying depending on the type of dairy product, pasteurization process, and storage conditions [3,40,70,100,103,123,128]. Xiao et al. [100] measured the content of free CML and protein-bound CML in 82 samples of seven dairy products (liquid milk, powdered milk, condensed milk, milk fat, cheese, ice cream, whey protein) using LC-MS/MS. The CML content in milk was higher in the bound form than in the free form, but no correlation was found between CML content and protein content. Furthermore, both free and protein-bound CML levels were often higher in pasteurized milk than in ultra-high-temperature (UHT) sterilized milk [100]. However, another study found that pasteurization did not significantly increase CML or Pyr levels, whereas UHT sterilization increased protein-bound CML and Pyr levels [103]. This discrepancy is likely due to differences in the comparison subjects [41]. Meanwhile, in powdered milk, CML content was also higher in the bound form than in the free form, but both were nearly equivalent in formula milk powder and modified milk powder. Furthermore, Zhang et al. [75] reported that CML, CEL, and MG-H1/H3 could be used as markers to evaluate the degree of Maillard reaction in dairy products for lactose-intolerant individuals. Furthermore, condensed milk contains large amounts of bound CML and Pyr compared to free forms [103]. The CML content in unsweetened condensed milk was higher than in sweetened condensed milk. This is likely because the sugar in sweetened condensed milk is a non-reducing sugar (e.g., sucrose), which contributes little to CML formation [100]. Additionally, the highest levels of both free CML and protein-bound CML were found in whey protein samples. This is likely because powdered protein drinks are rich in protein, making them prone to CML formation. No significant difference in CML content was observed between natural cheese and processed cheese. Furthermore, CML detected in ice cream manufactured at low temperatures is thought to originate from the raw materials [100]. Zhang et al. [131] measured nine types of AGEs in 17 dairy products. CML, CEL, GOLD, G-H1, MG-Hs, and others were detected in all samples, while MOLD, Arg-P, and Pent were scarcely detected. Furthermore, due to the effects of additives such as high-fructose/glucose syrup, casein and its derivatives, and phosphates, coffee creamer milk and flavored milk contained relatively high levels of AGEs [131].

### 4.3. Cereal and Bakery Products

Cereal products such as bread, cookies, tortillas, sweet pastries, pasta, and instant noodles are commonly consumed foods in daily life, and AGEs content has been measured in various cereal products [3,110,113,123,130,131]. The AGEs in these foods are primarily CML, CEL, F-Lys, Pyr, G-Hs, MG-Hs, GOLD, MOLD, and Pent. Their content varies depending on raw material components (proteins, amino acids, lipids, dietary fiber), processing time, and processing temperature. Cheng et al. [129] measured CML and CEL content in nine cereal products (chicken cookies, baby biscuits, white lotus seed paste mooncakes, almond biscuits, cookies, bread, sweet heart pastries, fried bread sticks, instant noodles) using UHPLC-QqQ-MS/MS. They found CML and CEL levels were significantly higher in bread and biscuits than in sweet heart pastries or instant noodles, with baby biscuits showing the highest values. Scheijen et al. [123] measured CML, CEL, and MG-H1 levels in 65 grain product samples (bread, potatoes, pastries, biscuits, cereals, pizza, etc.) using UPLC-MS/MS. MG-H1 was detected at higher concentrations than CML or CEL. Furthermore, Jost et al. [110] reported that AGEs such as CML, CEL, Pyr, G-H3, and MG-H1/3 were present in five types of bread (wheat, brown rice bread, rye bread, pumpernickel, crispbread), and that they increased during the baking process among the bread manufacturing steps (dough mixing, fermentation, baking). Treibmann et al. [105] quantified Lys-derived protein-bound AGEs (CML, CEL, F-Lys, MG-H1) in model cookies and commercial bakery products containing honey, banana, and invert sugar syrup. While no significant difference in CML formation was observed when glucose and fructose were added, the biscuit with the highest fructose content also showed the highest levels of CEL and MG-H1. Furthermore, increasing butter content promoted the formation of α-dicarbonyl compounds, which, in conjunction with the formation of short-chain α-dicarbonyl compounds via lipid oxidation reactions, led to increased protein-bound AGEs [89]. Whole egg liquid provided sufficient protein and free amino acids for baking butter biscuits. As the addition amount increased, the content of free CML and CEL initially increased and then decreased. This suggests that free CML and CEL participated in subsequent reactions as protein-bound CML and CEL gradually increased [95]. According to Zhang et al.’s [131] database, AGEs content in cereals and cereal products was relatively moderate, except for instant noodles and fried bread sticks. Breakfast cereals had higher AGEs content than other cereal products, particularly corn flakes and cocoa balls. This is thought to result from the use of added sugar and heating processes (such as extrusion or baking). Conversely, traditional Chinese staple foods like rice, noodles, dumplings, and steamed buns have low AGEs content, likely due to low-temperature cooking and minimal use of sugar and vegetable oils [131].

### 4.4. Fruits, Vegetables and Nuts

As Table 5 shows, fresh fruits and vegetables contain only trace amounts of AGEs, and there are few reports specifically focusing on these foods. Preservation processes like canning or drying (dried fruits classified as snacks) can significantly increase AGEs levels, likely due to heat treatment, high sugar content, and prolonged storage. Scheijen et al. [123] measured CML, CEL, and MG-H1 levels in four types of fruit and four types of cooked/processed vegetables (fried, stewed, canned). While MG-H1 levels reached tens of μg g^−1^ in some cases, CML and CEL were mostly below 1 μg g^−1^. Zhang et al. [67] also analyzed nine types of fruit and 38 types of vegetables (both raw and cooked). For vegetables, cooking method influenced AGEs formation: stir-frying tomatoes, broccoli, eggplant, onions, etc., increased AGEs levels by 1.2 to 2.4 times compared to steaming or boiling. Furthermore, olive pickles and cooked starchy plants like sweet potatoes showed the highest AGEs levels among vegetables. This is thought to result from the prolonged brining process and accelerated breakdown of starch into reducing sugars during processing [131].

Meanwhile, nuts and seeds are rich in AGEs [40], with G-H1 and MG-Hs sometimes reaching hundreds of μg g^−1^. The proteins, lipids, and carbohydrates in nuts correlate with the formation of various AGEs during heating processes like roasting. Zhang et al. [104] showed that in samples of raw and roasted almonds, the levels of bound CML and CEL were higher than those of free CML and CEL, and that roasting increased the levels of both simultaneously. Furthermore, Pyr was detected in all roasted almonds but not in raw almonds. Scheijen et al. [123] measured CML, CEL, and MG-H1 levels in 13 types of nuts and seeds, showing MG-H1 was present in higher amounts than CML or CEL. Furthermore, Zhang et al. [131] reported that nuts and seeds contained the highest levels of AGEs among various food categories. CML, CEL, G-H1, and MG-Hs were detected at high concentrations in these foods, while GOLD and Arg-P were detected in trace amounts. MOLD and Pent were scarcely detected. Furthermore, in nuts like peanuts and pecans, roasting and honey roasting dramatically increased MG-H levels, likely due to the combined effects of high glucose, unsaturated fatty acids, and intense heat treatment [131].

### 4.5. Beverages

Coffee, cocoa, green tea, black tea, and other beverages are major non-alcoholic drinks enjoyed worldwide. Since they undergo heat processing, their AGEs content is often measured. Coffee is typically processed by roasting, so roasting temperature and time affect the AGEs content of green coffee beans. Liu et al. [81] showed that CML content in coffee during roasting slowly increases for the first 10 min, then sharply decreases for the next 2 min, and subsequently increases again, reaching the lowest CML content after roasting at 235 °C for 12 min. Additionally, Loaec et al. [83] measured CML content in 24 commercial coffee substitutes and 12 instant coffees, showing a correlation with the samples’ protein content. Jiao et al. [97] measured CML and CEL content in 99 tea leaf samples from 14 regions (44 green tea, 7 oolong tea, 41 black tea, 7 dark tea), showing higher levels in black and dark teas and lower levels in green and oolong teas. These results suggest tea processing significantly affects CML and CEL content. The high CML and CEL content in black tea resulted from increased hydrolytic activity of protease, amylase, and invertase during the withering process, alongside moisture loss, leading to increased amino acids and monosaccharides—the active precursors of CML and CEL. In black tea, high CML and CEL content was likely due to the thermal effects of high-temperature, long-duration composting fermentation. Conversely, in green tea, heating processes like roasting, steaming, or microwave treatment inactivated enzymes. In oolong tea, lower levels of withering and fermentation, combined with higher catechin content in both green and oolong teas compared to black and dark oolong teas, are considered factors contributing to the lower CML and CEL content in these teas. AGEs content is generally low in common beverages like soft drinks, but tends to be higher in those containing more sugar and other components, such as fruit juices, caramel milk tea, and milk coffee [97,131]. Meanwhile, among alcoholic beverages, beer is widely consumed globally. During beer brewing process, reducing sugars and amino compounds in the raw materials react to form both free and bound AGEs. Hellwig et al. [107] measured F-Lys, Pyr, MG-H1, and Arg-P in different beer types (Pilsner, Dark, Bock, Wheat beer, Non-alcoholic beer) using LC-MS/MS. Notably, F-Lys was present at high concentrations in both free and bound forms, while Pyr and MG-H1 were also detected at relatively high levels. However, only trace amounts of free Arg-P were detected. Hellwig et al. [84] measured the content of free Pyr and peptide-bound Pyr in beverages fortified with whey protein hydrolysate (WPH), soy protein hydrolysate (SPH), and collagen protein hydrolysate (CPH). They reported that peptide-bound Pyr comprised 96% of the total Pyr.

### 4.6. Sauces, Condiments, and Other Processed Foods

Condiments are widely used worldwide to enhance flavor and umami during cooking. In particular, soy sauce and miso, which involve fermentation in their manufacturing processes, are prone to AGE formation. Li et al. [78] analyzed the free and bound CML content in soy sauces from Japan and China. Free CML was found to be higher in Chinese soy sauce, while bound CML tended to be higher in Japanese soy sauce. However, the free CML content in soy sauce was significantly higher than the bound CML content. Furthermore, fermented soy products and textured soy protein products had higher AGE levels, particularly CML, compared to unfermented or minimally processed products like tofu and soy milk. These results indicate that during fermentation, soy proteins and carbohydrates undergo partial hydrolysis into peptides, amino acids, and monosaccharides, leading to AGE formation via non-enzymatic browning. In fermented soy products like soy sauce, CML concentrations tend to increase with heat treatment and prolonged storage [40,78,108,131]. Furthermore, in soups, various sauces, and fats/oils, bound MG-H1 was present at higher levels than bound CML or CEL. While peanut sauce contained relatively high levels, tomato soup, mayonnaise, butter, and olive oil contained almost none [123].

## 5. Conclusions and Future Prospects

The accumulation of exogenous d-AGEs derived from food and endogenous AGEs produced in the body poses various health risks, and therefore it is important to reduce them. Particularly, AGEs in food, due to their ease of formation and the large quantities produced, are problematic when consumed in excess. However, as long as AGE precursors are always present in food, completely suppressing AGE formation during cooking and processing remains difficult. Furthermore, the Maillard reaction responsible for AGE formation is also involved in sensory qualities like flavor. Therefore, it is necessary to minimize AGE formation without sacrificing these qualities. The amount of AGEs formed in food depends on food components, storage methods, processing methods, and processing conditions. Therefore, it is necessary to control these factors and monitor AGEs levels in food, and to evaluate the intake of AGEs from these foods and their health effects. However, because multiple foods with varying AGEs levels are consumed simultaneously, accurately determining AGEs intake is difficult, and current understanding of the effects of AGEs intake at different levels is insufficient. Furthermore, it is not fully understood which of the various AGEs formed are more toxic, and therefore, a tolerable daily intake has not been established. Therefore, an urgent priority is to clarify the relationship between dietary AGEs intake and its adverse effects on human health, and to establish clear safety guidelines for consuming AGEs-containing foods.

To achieve this, it is necessary to clarify the relationship between the AGE content in food, biological exposure levels and the onset of toxicity, and it is essential to develop a simple and accurate method for measuring AGEs. AGEs in food exist as free forms and peptide/protein-bound forms, each with distinct chemical structures and properties, making simultaneous analysis using a unified method difficult. Consequently, efficient extraction, concentration, and separation methods, considering their stability, have been devised, and various analytical approaches have been reported. Foods vary in form—solid, semi-solid, liquid—and contain proteins, lipids, carbohydrates, and various low-molecular-weight components. Since AGEs also exhibit diverse properties, sample pretreatment is necessary to isolate and purify AGEs from complex matrices. When the sample contains high fat content, defatting using organic solvents like n-hexane is necessary. To separate free and bound forms, it is necessary to denature and precipitate the protein or utilize the molecular sieve effect to separate low-molecular-weight and high-molecular-weight components. In addition, AGEs bound within peptides or proteins exist in multiple forms depending on their constituent amino acid composition. Separating and measuring these individually is difficult, necessitating hydrolysis to release the AGEs. However, acid hydrolysis cannot be applied to AGEs unstable in acid, and enzymatic hydrolysis also has its drawbacks, such as requiring a long processing time. Furthermore, when considering other sample pretreatments besides hydrolysis—such as degreasing, reduction, and extraction—there is still no method that can perfectly address all aspects, including the specific AGEs targeted, operational simplicity and speed, and cost. Therefore, in order to simplify and streamline these complex and labor-intensive sample pretreatments, it is desirable to miniaturize the entire pretreatment process and to achieve high throughput and automation for multi-sample analysis. Recently, simple and environmentally friendly green sample preparation methods have been proposed, such as solid-phase microextraction (SPME), dispersive liquid microextraction (DLLME) [136], and ultrasound-assisted extraction (UAE) [11,137,138]. SPME can extract and concentrate compounds onto a fiber stationary phase with little use of organic solvents, improving detection sensitivity (Table 1) [127]. In particular, in-tube SPME [139], which uses a capillary column as an extraction device, is expected to be an effective sample pretreatment method because it can be coupled online with HPLC to automate sample extraction, concentration, separation, detection, and data analysis. Furthermore, DLLME is a liquid–liquid extraction using a minimal amount of solvent, and UAE is a non-thermal crushing extraction method using ultrasonic mechanical energy. Both are promising as efficient sample preparation methods. On the other hand, for methods to detect various AGEs, the ELISA method does not require large equipment and is relatively simple to operate. However, it requires specific antibodies against AGEs, has issues such as cross-reactivity, and only yields semi-quantitative results. Therefore, while it is suitable for screening, instrumental analysis is necessary to obtain more accurate measurements. Among instrumental analyses, LC-MS/MS is widely used as the most superior method, enabling the selective and highly sensitive simultaneous analysis of a wide variety of AGEs. Furthermore, the use of UHPLC-MS/MS has improved sample processing capacity by reducing solvent consumption and shortening analysis time. However, all methods face challenges such as matrix interference and the need to improve the resolution of structurally similar compounds. Additionally, the equipment is expensive and requires significant maintenance costs. Therefore, developing sample pretreatment methods that can selectively and efficiently separate and purify AGEs from the sample matrix to reduce the burden on the analytical instrument, along with easy integration with the analyzer, will be important future challenges.

Thus, advances in AGE analytical methods have revealed AGE levels in various foods, leading to the development of cooking methods that reduce AGE content in foods and improvements in dietary habits to reduce intake of foods high in AGEs. For example, it has been demonstrated that cooking foods in a moist state, shortening cooking time at high temperatures while cooking at lower temperatures, and adding compounds that inhibit AGE formation—such as acidic components like lemon juice or vinegar, or antioxidant components—can significantly reduce d-AGE formation [8]. Furthermore, in processed food manufacturing, controlling food composition, processing methods, and storage conditions can also reduce AGEs content. Therefore, to assess human health risks, it is necessary to build an AGEs database with clear intake guidelines covering a wide range of food categories. Comprehensive databases of dietary AGEs based on food consumption and dietary habits have been reported [123,131], but they cannot be considered exhaustive due to limitations in detectable AGEs. Furthermore, only 10% to 30% of AGEs are absorbed into systemic circulation after food intake. Of the absorbed AGEs, 90% of free AGEs and 60% of bound AGEs are excreted from the body, meaning only 7% of AGEs accumulate within the body [18,40]. Therefore, when considering the health effects of d-AGEs, while understanding the AGEs content in foods is essential, it is also crucial to evaluate the actual exposure levels of AGEs ingested through diet and their potential harm. Furthermore, traditional Japanese seasonings such as miso and soy sauce are high in AGEs [78,108], and despite many Japanese people ingesting AGEs daily through their diet, Japanese have one of the world’s longest-lifespans. This may be because miso and soy sauce are both high in AGEs and high in melanoidins, and the antioxidant properties of melanoidins, among other physiological activities, may suppress the effects of AGEs [140]. Furthermore, melanoidins found in coffee, cocoa, and bread have been reported to act as scavengers for α-dicarbonyl compounds, which are precursors to AGEs [141]. Therefore, evaluating the health effects of foods by focusing solely on their AGE content can lead to an incorrect understanding of the function of the food itself, so it is important to comprehensively evaluate them, including coexisting components. Furthermore, while long-term excessive consumption of foods high in AGEs is problematic, this suggests that health risks can be reduced by simultaneously consuming foods that break down or inhibit the absorption of AGEs during intake.

As discussed, although dietary exogenous AGEs contribute little to in vivo accumulation, they are rapidly and abundantly generated in foods during heating and processing and are consumed daily. Therefore, their health impacts must be considered similarly to endogenous AGEs, which are slowly generated and accumulate within the body. Therefore, it is crucial to devise strategies to suppress AGEs formation and to neutralize, metabolize, or break down formed AGEs, regardless of whether they are exogenous or endogenous. However, the structure, function, and biological effects of AGEs in food and within the body remain poorly understood in many aspects. Consequently, developing effective methods to measure and evaluate them is a critical future challenge. In particular, establishing standardized methods to facilitate easy comparison of measurement data between laboratories is an urgent issue, and clarifying the relationship between these analytical outcomes and toxicological endpoints is crucial. Finally, we hope the content of this review will inspire scientists to pursue intriguing research themes, leading to further innovative ideas and the creation of new technologies to address various challenges.

## Figures and Tables

**Table 1:** Sample preparation and analytical techniques for the analysis of AGEs in various foods.

Food Sample *	Sample Preparation	Detection	Analytical Column	AGEs	LOD	LOQ	Recovery	Ref.
Powdered and liquid infant formulas	Defatting and precipitation with water/CHCl_3_/MeOH (0.8/2/1, *v*/*v*/*v*), reduction with NaBH_4_/borate buffer at 4 °C for 6 h, hydrolysis with 6 M HCl at 110 °C for 18 h, derivatization with SOCl_2_ in MeOH and TFA.	GC-MS	DB5-MS (30 m × 0.25 mm, 0.25 μm)	CML	0.1 μg/g protein	1.0 μg/g protein		[67]
Fried fish nuggets (180 °C, 4–6 min)	Defatting with CHCl_3_:acetone (1:3, *v*:*v*), reduction with NaBH_4_/borate buffer at 4 °C overnight, hydrolysis with 6 M HCl at 110 °C for 24 h, derivatization with SOCl_2_ in MeOH and TFA.	GC-MS	DB5-MS capillary (30 m × 0.25 mm, 0.25 μm)	CML				[68]
Roasted nuts	Defatting with n-hexane/MeOH (2/1, *v*/*v*), hydrolysis with 6 M HCl at 110 °C for 18 h, derivatization with SOCl_2_ in MeOH and TFA.	GC-MS/MS	DB5-MS (30 m × 0.25 mm, 0.25 μm)	CML		0.3–0.9 μg/g dry matter		[69]
Infant formulas (65 °C; 48 days)	Enzymatic hydrolysis using pepsin, Pronase E, leucine aminopeptidase and prolidase at 37 °C for 72 h.	HPLC- UV	SphereClone™ ODS (4.6 × 250 mm, 5 μm)	Pyr				[70]
UHT milk (22 °C; 12 months)	Enzymatic hydrolysis using pepsin, Pronase E, leucine aminopeptidase and prolidase at 37 °C for 72 h.	UHPLC-DAD	HSS T3 (2.1 × 100 mm, 1.8 μm)	Pyr	0.5 μg/g protein			[71]
Raw and cooked meat (fried, boiled and baked)	Defatting with CHCl_3_:MeOH (2:1, *v*:*v*), reduction with NaBH_4_/borate buffer at room temp. for 4 h, hydrolysis with 4 M HCl at 110 °C for 20 h, derivatization with OPA.	HPLC-FLD	TSK gel ODS-80 TM (4.6 × 250 mm, 5 µm)	CML	1.5 ng/mL	5.0 ng/mL	102.2–104.5%	[72,73]
Grilled, fried, and baked beef	Reduction with NaBH_4_/borate buffer at room temp. for 4 h, hydrolysis with 6 M HCl at 110 °C for 20 h, SPE with Oasis HLB.	UPLC-MS	Zorbax SB-C18 (2.1 × 50 mm, 1.8 µm)	CML	1 ng/mL, 0.33 μg/g protein	10 ng/mL 3.3 μg/g protein	84–134%	[74]
Conventional UHT milk, Lactose-hydrolyzed UHT milk	Free AGEs: Deproteinization with MeOH. Total AGEs: Microwave assisted acid hydrolysis with 6 M HCl at 150 °C for 1 min and 165 °C for 10 min. For Pyr: Enzymatic hydrolysis with multi enzyme (pepsin, Pronase E, leucine aminopeptidase, prolidase) at 37 °C for 24 h.	UHPLC-MS	BioZen Peptide XB-C18 LC (2.1 × 150 mm, 1.7 μm)	CML CEL GO-H1/H3 G-H2 MG-H1/3 MG-H2 GOLD MOLD GOLA GALA Pent Arg-P Pyr CMC				[75]
Beer, Sausage	Lyophilize and dilute with 0.01 M HCl (defatting of sausage samples with n-hexane), enzymatic hydrolysis with 4 types of enzyme (pepsin, pronase E, leucine aminopeptidase, prolidase), derivatization DNS-Cl.	UPLC-DAD-MS	Acquity UPLC BEH C18 (2.1 × 100 mm, 1.8 μm)	CML CEL G-H1 G-H2 G-H3 MG-H1 MG-H2 MG-H3 MOLD GOLD Pyr LM GALA 2-SC	3.2 ng/mL 8.7 14.2 5.2 35.6 3.4 4.0 24. 1.0 20.6 9.6 43.3 4.0 20.2	9.8 ng/mL 26.3 43.0 15.9 107.8 10.2 12.2 72.6 3.0 62.5 29.2 131.3 12.1 61.3	93–107% 94–112 93–123 83–125 66–141 93–110 78–105 96–136 80–104 58–117 83–110 81–115 88–119 84–113	[76]
Dairy products (raw milk, pasteurized milk, skimmed milk, UHT milk, condensed milk, half cream milk)	Reduction with NaBH_4_/borate buffer at room temperature for 4 h, acid hydrolysis with 6 M HCl at 110 °C for 24 h, SPE with Oasis HLB. Enzymatic digestion with multi enzyme (pepsin, Pronase E, leucine aminopeptidase, prolidase) at 37 °C, SPE with Oasis HLB.	LC-MS/MS	Atlantis dC18 (2.1 × 150 mm, 3 μm)	CML	8 μg/g protein	27 μg/g protein	91–107%	[77]
Japanese and Chinese soy sauce (12)	Free CML: (A) Dilution (600-fold) with MeOH:water (1:9, *v*/*v*); (B) Deprotenization with methanol (75%) and dilution (600-fold) with MeOH:water (1:9, *v*/*v*); (C) C18 SPE column treatment; (D) Deprotenization with MeOH (75%) and C18 SPE Protein bound CML: Reduction with NaBH_4_/borate buffer at 4 °C for 12 h, removed free CML, hydrolysis with 6 M HCl at 110 °C for a certain time, SPE with C18.	LC-MS/MS	Atlantis C18 (4.6 × 150 mm, 5 μm)	CML			Free: 98.6–98.7%	[78]
Cookies (okara, cellulose c, pea fiber, chitosan)	Reduction with NaBH_4_/borate buffer at room temp. overnight, hydrolysis with 6 M HCl at 110 °C for 24 h.	LC-MS/MS	TSK Gel Amide-80 (2 × 250 mm, 5 μm)	CML				[79]
Chocolate-flavored drink, malt drink mix (27)	Reduction with NaBH_4_/borate buffer at room temp. for 4 h, defatting and precipitation with water/ethanol/CHCl_3_ (1:2:4, *v*/*v*), hydrolysis with 6 M HCl at 110 °C for 20 h, SPE with C18.	LC-IT-MS/MS	Hypercarb (2.1 × 100 mm, 5 μm)	CML	33.6 ng/g protein	102.0 ng/g protein	100–111%	[80]
Coffee beans under different roasting conditions	Defatting with petroleum ether, reduction with NaBH_4_/borate buffer overnight at 4 °C, precipitation with chloroform/methanol (2:1, *v*/*v*), hydrolysis with 6 M HCl at 160 °C for 35 min, SPE with Oasis MCX.	LC-IT-MS/MS	Zorbax SB-C18 (2.1 × 50 mm, 1.8 μm),	CML				[81]
Coffee substitutes and instant coffees (36)	Reduction with NaBH_4_/borate buffer at room temp. for 4 h, hydrolysis with 6 M HCl at 110 °C for 20 h.	LC–MS/MS	Hypercarb (2.1 × 100 mm, 5 μm)	CML	3 ng/g	9 ng/g		[82]
Food items (20) (nuggets, fried chicken, fried bacon, cheese, yogurt, tortilla, potatoes, pepper)	Reduction with NaBH_4_/borate buffer overnight at 4 °C, precipitation with HClO_4_, defatting with CHCl_3_-MeOH (2:1, *v*/*v*), hydrolysis with 6 M HCl at 110 °C for 20 h, derivatization with ethyl chloroformate, SPE with C18.	LC-IT-MS/MS	Kinetex C18 (3 × 150 mm, 2.6 μm)	CML				[83]
Protein hydrolysate enriched drinks (27)	Free: SPE with Cleanert PEP-2 SPE cartridge Peptide bound: complete enzymatic hydrolysis, SPE with Cleanert PEP-2 SPE.	UPLC-UV-MS/MS	Zorbax SB-C18 (2.1 × 150 mm, 5 μm),	Pyr	30.4 ng/mL	70.3 ng/mL	93.2–98.5%	[84]
Powdered gruel, Powdered infant formula	Free AGEs: Extract with water: MeOH (1:3, *v*/*v*), deproteinization with NFPA, SPE with TELOS neo PCX. Total AGEs: Hydrolysis with 6 M HCl at 110 °C for 24 h, SPE with TELOS neo PCX.	LC-MS/MS	Genesis Lightn AQ (4.6 × 250 mm, 4 μm)	CML CEL		5 ng/mL 5	82% 91	[85]
Cereal foods (bread crust, bread crumbs, biscuits, fried dough sticks)	Defatting with n-hexane, reduction with NaBH_4_/borate buffer at 4 °C for 12 h, precipitation with TCA, hydrolysis with 6 M HCl at 110 °C for 24 h, SPE with Styre Screen SSDBX.	LC-MS/MS	Xselect, HSS T3, RP18 (4.6 × 150 mm, 5 μm)	CML CEL		4 ng/g 3		[86]
Infant formula, Soybean products (80)	Hydrolysis with 6 M HCl at 110 °C for 20 h, SPE with Oasis HLB.	LC–MS/MS	Kinetex C18 (2.6 × 100 mm, 2.6 μm)	CML CEL	0.5 ng/g 5	1 ng/g 5	91.1% 84.2%	[87,88]
Ground beef, Raw and cooked eggs (32)	Reduction with NaBH_4_/borate buffer at 4 °C for 8 h, defatting and precipitation with CHCl_3_-MeOH (2:1, v:v), hydrolysis with 6 M HCl at 110 °C for 24 h, SPE with MCX cartridge.	LC-MS/MS	Atlantis HILIC silica (2.1 × 150 mm, 3 μm)	CML CEL	4 ng/g 5	12 ng/g 15	78–98% 81–108	[89,90]
Raw and sterilized meats, Fish muscle	Free form: deproteinization with 5% TCA, defatting with n-hexane, SPE with Oasis MCX. Bound form: reduction with NaBH_4_/borate buffer at 4 °C for 8 h, precipitation and defatting with CH_2_Cl_2_/MeOH (2:1, *v*/*v*), hydrolysis with 6 M HCl at 110 °C for 24 h, SPE with Oasis MCX.	LC-MS/MS	Atlantis HILIC silica (2.1 × 150 mm, 3 μm)	CML CEL	4 ng/g 5	12 ng/g 15	Free 101–122% Bound 79–105	[91,92]
Raw and sterilized pork	Defatting with n-hexane, reduction with NaBH_4_/borate buffer at 4 °C for 8 h, hydrolysis with 6 M HCl at 110 °C for 24 h, SPE with Oasis MCX.	LC-MS/MS	Atlantis silica HILIC ( 2.1 × 150 mm, 3 µm)	CML CEL	0.27 ng/mL	0.91 ng/mL	72–103% 71–116	[93]
Canned fishes, Cheeses, Cookies	Defatting with MeOH/CHCl_3_ (1:2, *v*/*v*), Free form: SPE with Oasis MCX, Bound form: reduction with NaBH_4_/borate buffer at 4 °C overnight, hydrolysis with 6 M HCl at 110 °C for 24 h, SPE with Oasis MCX.	LC-MS/MS	Hydro-RP 80A LC (2 × 150 mm, 4 μm)	CML CEL				[94,95,96]
Tea (99) (green teas, oolong teas, black teas, dark teas)	Defatting with n-hexane, reduction with NaBH_4_/borate buffer overnight at 4 °C, hydrolysis with 6 M HCl at 110 °C for 24 h, SPE with Oasis MCX.	LC–MS/MS	X-Bridge C18 (2.1 × 100 mm, 3.5 μm)	CML CEL	3.5 ng/mL 1.7	8.5 ng/mL 4.3	96–113% 96–121	[97]
Cookies	Free form: reduction with NaBH_4_/borate buffer at 4 °C overnight, deproteinization with 10% TCA, SPE with Oasis MCX. Bound form: precipitation with 10% TCA, hydrolysis with 6 M HCl at 110 °C for 24 h, SPE with Oasis MCX.	LC-MS/MS	X-Bridge C18 (2.1 × 100 mm, 3.5 μm)	CML CEL	4.71 ng/g 4.84	9.41 ng/g 9.68		[98]
Milk (LTLT, HTST, UHT and BS)	Defatting with n-hexane, reduction with NaBH_4_/borate buffer overnight at 4 °C, hydrolysis with 6 M HCl at 110 °C for 24 h, SPE with Oasis MCX.	LC-MS/MS	Waters T3 (2.1 × 150 mm, 3.5 µm)	CML CEL	44 ng/g 39	150 ng/g 130		[99]
Commercial dairy products (82) (liquid, powder, milk fats, condensed, ice cream, cheese, whey protein)	Defatting with n-hexane, reduction with NaBH_4_/borate buffer at 4 °C for 6 h, precipitation with 60% TCA, hydrolysis with 6 M HCl at 110 °C for 24 h, SPE with HiCapt MCX.	LC-MS/MS	Symmetry C18 (4.5 × 150 mm, 5 μm)	CML CEL	0.03 ng/g	0.1 ng/g	104–115%	[100]
Whole-milk powder (LTLT, HTST, ESL, UHT and BS)	Defatting with n-hexane, reduction with NaBH_4_/borate buffer at 4 °C overnight, hydrolysis with 6 M HCl at 110 °C for 24 h, SPE with Oasis MCX.	LC-MS/MS	Water T3 (2.1 × 150 mm, 3.5 µm)	CML CEL Pyr	44 ng/g 39 70	150 ng/g 130 240	95–105% 96–104 96–104	[101]
Milk	Reduction with NaBH_4_/borate buffer at room temp. for 2 h, precipitation with 20% TCA, hydrolysis with 6 M HCl at 110 °C for 23 h.	LC-MS/MS	Cortecs C18 (2.1 × 50 mm, 1.6 μm)	CML CEL Pent				[102]
Milk (raw, pasteurized, skimmed, UHT, condensed, half cream)	Free AGEs: deproteinized with acetone/MeOH (1:1, *v*/*v*), defatting with n-hexane, SPE with Oasis HLB. Bound form: reduction with NaBH_4_/borate buffer at room temp. for 4 h, hydrolysis with 6 M HCl at 110 °C for 24 h, SPE with Oasis HLB. Total AGEs: digestion with multi enzyme (pepsin, Pronase E, leucine aminopeptidase, prolidase) at 37 °C for 24 h, SPE with Oasis HLB.	LC-MS/MS	Atlantis dC18 (2.1 × 150 mm, 3 µm)	CML F-Lys Pyr		μg/g protein Free 0.04 0.03 0.01 Bound 0.28 1.09 33.03		[103]
Raw and roasted almonds and nuts (pistachios, almonds, peanuts)	Free AGEs: extraction with 1% NFPA, SPE with Strata-X-C. Free + bound AGEs: defatting with n-hexane, enzymatic hydrolysis using pepsin (24 h), pronase E (24 h), aminopeptidase and prolidase (24 h) at 37 °C.	LC-MS/MS	Prodigy ODS (2.0 × 150 mm, 5 μm)	CML CEL Pyr Arg-P Pent	3 ng/g 4 6 9 12	9 ng/g 12 16 28 38	92–106% 87–121 87–110 84–121 91–109	[104]
Bakery products (dough, baked cookies, purchased bakery products)	Defatting with petroleum ether, dialysis of precipitate with water, enzymatic hydrolysis using pepsin (24 h), Pronase E (24 h), leucine aminopeptidase and prolidase (48 h) at 37 °C.	LC-MS/MS	Zorbax SB C-18 (2.1 × 50 mm, 3.5 μm)	F-Lys Gl-Lys CML CEL MG-H1	2.1 μg/g 1.3 0.6 0.3 1.0	5.2 μg/g 3.7 1.1 0.7 2.6		[105]
Manuka honey	Dialysis, precipitation with TCA, hydrolysis with 6 M HCl at 110 °C for 23 h. Enzymatic hydrolysis with pepsin (24 h), Pronase E (24 h), leucine aminopeptidase and prolidase (24 h) at 37 °C.	LC-MS/MS	Zorbax 100 SB-C18 (2.1 × 50 mm, 3.5 μm)	CML CEL MG-H1 Formyline Pyr F Lys	0.15 ** 0.9 0.16 0.04 0.07 1.8	1.9 ** 4.0 0.55 0.12 0.23 5.1		[106]
Beer (52) (pilsner beer, dark beer, bock beer, wheat beer, alcohol-free beer)	Free form: membrane filtration (0.45 μm) without further workup.	LC-MS/MS	Zorbax 100 SB-C18 (2.1 × 50 mm, 3.5 μm)	F-Lys M-Lys Pyr Formyline Maltosine MG-H1 Arg-P	2.5 ng/mL 8.2 0.4 0.1 0.6 1.6 0.1	9.0 ng/mL 28.5 2.8 0.4 2.3 6.0 0.4		[107]
Beer (52) (pilsner beer, dark beer, bock beer, wheat beer, alcohol-free beer)	Bound form: dialysis at 4 °C for 4 days, lyophilization, enzymatic hydrolysis using pepsin, Pronase E, leucine-aminopeptidase at 37 °C for each 24 h, or acid hydrolysis with 6 M HCl at 110 °C for each 23 h.	LC-MS/MS	Zorbax 100 SB-C18 (2.1 × 50 mm, 3.5 μm)	F-Lys M-Lys Pyr Formyline Maltosine MG-H1 Arg-P	ng/mg protein 6.3 22.8 0.6 0.1 0.3 3.8 -	ng/mg protein 2.1 75.9 2.1 0.2 1.1 12.5 -		[107]
Soy sauce and beer(draft, dark, low-malt, beer-like alcoholic beverage)	Deproteinization with sulfosalicylic acid, SPE with Agilent C18.	LC-MS/MS	Intrada Amino Acid (2.0 × 100 mm, 3 μm)	CML CEL G-H1 MG-H1 GOLD MOLD Pent	2 ng/mL 2 4 2 0.2 1 9	7 ng/mL 5 12 7 0.7 5 30	85–112% 96–107 - 70–123 - - -	[108]
Raw pork, roast pork	Defatting with petroleum ether, homogenization with water/MeOH (81/19, *v*/*v*), protein precipitation by centrifugation, reduction with NaBH_4_/NaOH at room temp. for 1 h, hydrolysis with 6 M HCl at 110 °C for 20 h.	LC-MS/MS	Xselect, HSS T3, (3.0 × 250 mm, 5 μm)	CML GALA CEL GOLD GLA GOLA MOLD MGLA MOLA GODIC MODIC	nmol/g protein 0.26 0.22 0.27 0.57 0.69 3.58 1.11 0.75 1.75 0.71 0.39	nmol/g protein 0.77 0.65 0.80 1.72 2.06 10.74 3.34 2.24 5.26 2.12 1.16		[109]
Bread (raw dough, pre-proofing, fermentation, prebaking, baking) (wheat, brown bread, rye bread, pumpernickel, and crispbreads)	Reflux in water/2-propanol (1:1, *v*/*v*) containing 0.25 mL of thioglycerol for 3 h, protein precipitation and lyophilization. Acid hydrolysis: reduction with NaBH_4_/NaOH at room temp. for 1 h, hydrolysis with 6 M HCl at 110 °C for 20 h. Enzymatic hydrolysis: Pronase E (24 h), leucine aminopeptidase (24 h), and carboxypeptidase Y (24 h) at 37 °C, ultrafiltration (MW 3000).	LC-MS/MS	Xselect HSS T3 (3.0 × 250 mm, 5 μm)	CEL CEA MG -H1 MG-H3 CML GALA CMA G-H3 Pyr	0.05 μg/g 0.07 0.05 0.06 0.06 0.06 0.17 0.11 0.12	0.15 μg/g 0.24 0.18 0.16 0.21 0.19 0.57 0.37 0.40		[110]
Meat and fish, meat dishes, cereals, pasta, sweets, fruits, vegetables, sauces	Reduction with NaBH_4_/borate buffer overnight at 4 °C, precipitation with CHCl_3_-MeOH (2:1, *v*/*v*)), hydrolysis with 6 M HCl at 110 °C for 24 h, SPE with C18.	UPLC–MS/MS	Acquity UPLC BEH C18 (2.1 × 50 mm, 1.7 μm)	CML	0.5 μg/g protein	1.6 μg/g protein		[111]
Sterilized milk	Reduction with NaBH_4_/borate buffer at 4 °C overnight, hydrolysis with 6 M HCl at 110 °C for 24 h, SPE with Oasis MCX.	UPLC-MS/MS	Acquity BEH Amide (2.1 × 100 mm, 1.7 μm)	CML	0.05 μg/g sample	0.15 μg/g sample	97–98%	[112]
Processed foods (54) (fried and baked foods, infant formulas, protein powder)	Defatting with n-hexane, reduction with NaBH_4_/borate buffer overnight at 4 °C, precipitated with TCA, hydrolysis with 6 M HCl at 110 °C for 24 h, derivatization with FMOC-Cl, SPE with Oasis HLB.	UPLC–MS/MS	Acquity UPLC BEH C18 (2.1 × 100 mm, 1.7 μm)	CML CEL	2 ng/mL 2	7 ng/mL 7	102–112% 86–114	[113]
Bread crust	Reduction with NaBH_4_/borate buffer at 20 °C for 4 h, hydrolysis with 8 M HCl at 110 °C for 23 h.	UPLC-MS/MS	Atlantis T3 (4.6 × 150 mm, 3 μm)	CML CEL	0.75 ng/g	2.5 ng/g		[114]
Roasted beef patties	Defatting with n-hexane, reduction with NaBH_4_/borate buffer at room temp. for 4 h, hydrolysis with 8 M HCl at 110 °C for 24 h, SPE with Waters MCX.	UPLC-MS/MS	HSS T3 (2.1 × 150 mm, 3.5 μm)	CML CEL	52 ng/g 98	104 ng/g 392		[115]
Milk powder and reconstituted milk (LT, HT, ESL, UHT, CS)	Defatting with n-hexane, reduction with NaBH_4_/borate buffer at 4 °C for 12 h, hydrolysis with 6 M HCl at 110 °C for 24 h, SPE with Oasis MCX.	UPLC-MS/MS	T3 (2.1 × 150 mm, 3.5 µm)	CML CEL	44 ng/mL 39	150 ng/mL 130	95–105% 96–104	[116]
Roasted beef patties	Free AGEs: Defatting with n-hexane, deproteinization with TCA, SPE with Cleanert PCX. Protein-bound AGEs: Reduction with NaBH_4_/borate buffer at 4 °C for 12 h, hydrolysis with 6 M HCl at 110 °C for 24 h, SPE with MCX.	UPLC-MS/MS	HSS T3 (2.1 × 150 mm, 3.5 μm)	CML CEL	Free 0.63–1.65 ng/g Bound 8.8–30.1 ng/g	Free 1.31–4.47 ng/g Bound 26.3–89.4 ng/g		[117]
Raw and heated pork	Free form: deproteinization with 5% TCA, defatting with n-hexane, SPE with Oasis MCX. Bound form: reduction with NaBH_4_/borate buffer at 4 °C for 8 h, precipitation with MeOH-CHCl_3_ (1:2, *v*/*v*), hydrolysis with 6 M HCl at 110 °C for 24 h, SPE with Oasis MCX.	UPLC- MS/MS	Acquity UPLC HSS T3 (2.1 × 100 mm, 1.8 μm)	CML CEL	0.27 ng/mL	0.91 ng/mL		[118]
Plant-based burgers	Defatting with n-hexane, reduction with NaBH_4_/borate buffer at 25 °C for 4 h, hydrolysis with 6 M HCl at 110 °C for 24 h.	UHPLC-MS/MS	X-Bridge C18 (2.1 × 100 mm, 3.5 μm)	CML CEL	1.17 ng/mL 1.25	9.41 ng/mL 40	95.6% 107.1	[119]
Sausage	Extraction with MeOH:water (3:1), deproteinization with NFPA, SPE with Oasis MCX cartridge, derivatization with OPD.	UPLC-MS/MS	Synergi Hydro–RP 80 Å (2 × 250 mm, 4 µm)	CML CEL				[120]
Stewed meat products (beef, pork, mutton)	Deproteinization with 80% CH_3_CN-water, defatting with n-hexane, SPE with HyperSep SCX.	UPLC-MS/MS	HSS T3 (2.1 mm × 100 mm, 1.8 μm)	CML CEL	16 ng/g 16	50 ng/g 50	75–111% 74–113	[121]
Whey proteins	Hydrolysis with 6 M HCl at 110 °C for 20 h.	UHPLC-MS/MS	Kinetex HILIC (2.1 × 100 mm, 2.6 μm)	CML CEL MG-H1	0.5 ng/mL 0.5 0.7	2.5 ng/mL 2.0 3.0		[122]
Selected food items (222) (meat and fish products, eggs, milk, cheese, yogurt, bread, cereal, fruits, vegetables, nuts, beverages, sauces, condiments)	Reduction with NaBH_4_/borate buffer at room temp. for 2 h, defatting and precipitation with CHCl_3_-MeOH (2:1, *v*/*v*), hydrolysis with 6 M HCl at 110 °C for 24 h.	UPLC–MS/MS	Acquity UPLC BEH C18 (2.1 × 50 mm, 1.7 μm)	CML CEL MG-H1		0.2 μg/g 0.2 5.0		[123]
Fish cakes, Fried shrimp	Defatting and precipitation with CHCl_3_-acetone (1:3, *v*/*v*), reduction with NaBH_4_/borate buffer at 4 °C for 8 h, hydrolysis with 6 M HCl at 110 °C for 24 h, SPE with MCX.	UPLC-MS/MS	Acquity BEH Amide (2.1 × 100 mm, 1.7 μm)	CML CEL MG-H1				[124,125]
Milk powder	Free form: Deproteinization with 1% TCA, defatting with n-hexane, SPE with MCX.	UHPLC-MS/MS	Acquity BEH C18 (2.1 × 50 mm, 1.7 μm)	CML CEL Pyr	4 ng/g 4 19	12 ng/g 12 58	92–98% 93–102 86–91	[126]
Milk powder	Bound form: Precipitation with 1% TCA, reduction with NaBH_4_/borate buffer at 4 °C overnight, hydrolysis with 6 M HCl at 110 °C for 24 h, SPE with MCX.	UHPLC-MS/MS	Acquity BEH C18 (2.1 × 50 mm, 1.7 μm)	CML CEL Pyr	20 ng/g 20 95	60 ng/g 60 290	90–107% 85–91 81–86	[126]
Chicken and fish products (boiled, canned, roast, fried)	Free AGEs: deproteinization with TCA, defatting with n-hexane, SPME with BrCOF fiber. Bound AGEs: defatting with n-hexane, reduction with NaBH_4_/borate buffer at 4 °C for overnight, hydrolysis with 6 M HCl at 110 °C for 24 h, SPME with BrCOF fiber.	UPLC- MS/MS	Kinetex Biphenyl (2.6 μm, 150 mm × 4.6 mm)	CML CEL Pyr	0.001 ng/mL 0.001 0.005	0.005 ng/mL 0.005 0.008	89–114% 88–117 85–112 87–116% 89–113 88–113	[127]
UHT milk (22 °C; 12 months)	Microwave assisted acid hydrolysis with 6 M HCl at 150 °C for 1 min and 165 °C for 10 min.	UHPLC-MS/MS	BioZen Peptide XB-C18 (2.1 × 150 mm, 1.7 μm)	CML CEL G-H1/3 G-H2 MG-H1/3 MG-H2				[71]
Canned meat and seafood product (pork, chicken, spam, snail, saury, eel, mackerel, tuna, sardine, clam)	Defatting with CH_2_Cl_2_/n-hexane (4:1, *v*/*v*), reduction with NaBH_4_/borate buffer at room temp. for 4 h, hydrolysis with 4 M HCl at 110 °C for 20 h.	UHPLC-MS/MS	Acquity BEH amide (2.1 × 100 mm, 1.7 μm)	CML CEL G-H1 MG-H1 MOLD GOLD Arg-P	0.15 ng/mL 0.15 0.30 1.22 4.88 2.44 0.04	0.30 ng/mL 0.30 0.61 2.44 9.77 4.88 0.15	106.7% 89.5 92.4 95.5 97.0 118.6 118.7	[128]
Baked and fried foods (cookies, biscuit, mooncakes, sweetheart pastry, fried breadstick, instant noodles)	Sonication with acetone and separation to supernatant (free) and precipitate (bound), reduction of precipitate with NaBH_4_/borate buffer at room temp. for 4 h, hydrolysis with 6 M HCl at 110 °C for 24 h, SPE with Oasis MCX, mix free and bound fractions.	UHPLC-QqQ-MS/MS	Acquity HSS T3 (2.1 × 100 mm, 1.8 μm)	CML CEL	0.94 μg/g 0.86	1.91 μg/g 1.64	90–108% 89–106	[129]
Milk, infant formula, cereal, nuts and meat products (cookies, corn flakes, pretzel sticks, chips, peanuts, cashews, hazelnuts, salami sticks)	Reduction with NaBH_4_/borate buffer at room temp. for 4 h, microwave assisted acid hydrolysis with 6 M HCl.	UHPLC-OrbiTrap Q Extractive MS	Acquity BEH amide (2.1 × 100 mm, 1.7 µm)	CML CEL MG-H1 MG-H3 GO-H1 GOLD Arg-P	4.35 ng/mL 1.89 5.36 5.42 1.66 12.71 1.56	13.18 ng/mL 5.72 16.25 16.44 5.03 38.52 4.71	105% 94 103 108 84 97 -	[130]
Commonly consumed foods (334) (meat products, fish, seafoods, eggs, milk, yogurt, cheese, bread, pastry, biscuits, sweets, snacks, cereals, fruits, vegetables, nuts, seeds, beverages, soy products, sauces, condiment)	Precipitation with CHCl_3_-MeOH (2:1, *v*/*v*), reduction with NaBH_4_/borate buffer at 25 °C for 3 h, hydrolysis with 6 M HCl at 110 °C for 24 h, SPE with Oasis MCX.	UHPLC-QqQ-MS/MS	Kinetex C18 (3.0 × 100 mm i.d., 2.6 μm)	CML CEL GOLD G-H1 MOLD MG-H2 MG-H1/3 Pent Arg-P	3.75 ng/mL 5.21 13.57 7.28 4.78 10.45 13.83 19.76 1.59	11.25 ng/mL 15.60 40.74 21.84 14.34 31.35 41.49 59.28 4.77	90–92% 86–101 91–111 88–90 87–89 99–111 89–96 89–107 87–92	[131]
Roasted pork and chicken, UHT milk	Defatting with CH_2_Cl_2_/MeOH (8:2 *v*/*v*), microwave assisted acid hydrolysis with 6 M HCl at 150 °C for 1 min and 165 °C for 10 min.	UHPLC-MS/MS (OrbiTrap Q Exactive MS)	BioZen Peptide XB-C18 (2.1 × 150 mm, 1.7 μm)	CML CEL G-H1/3 G-H2 MG-H1/3 MG-2 Arg-P GOLD MOLD GOLA GALA Pent	2.64 ng/mL 1.77 3.45 5.19 3.9 3.6 1.26 8.79 9.72 3.21 2.64 3.09	7.92 ng/mL 5.34 10.35 15.6 11.67 10.83 3.81 26.37 29.19 9.63 7.89 9.27	42–101% 76–112 58–113 83–90 75–106 75–110 76–115 61–103 67–129 72–104 88–125 50–103	[132]
Powdered formula	Digestion with Proteinase K at 37 °C for 2 h.	ELISA		CML				[85]
Food items (20)	Digestion with Proteinase K.	ELISA	CML-AGE ELISA kit	CML				[83]
Raw and boiled broiler muscle		ELISA	Echelon Biosciences CML ELSA kit	CML				[133]
Nutritional milk formulas		ELISA	CML ELSA kit	CML				[134]
Fried meat products	Homogenization, digestion with proteinase K.	ELISA	OxiSelect^TM^ ELSA Kit	CML CEL	16 ng/g 16 ng/g			[135]

* The numbers in parentheses indicate the number of sample species. ** LOD & LOQ: mmol/mol Leu. Abbreviations: CML, *N*^ε^-(carboxymethyl)lysine; CEL, *N*^ε^-(carboxyethyl)lysine; F-Lys, fructosyl-Lys; G-Lys, glucosyl-Lys; M-Lys, maltulosyl-Lys; Pyr, pyrraline; GLAP, glyceraldehyde-derived pyridinium compound; G-Hs (G-H1/H2/H3), glyoxal-hydroimidazolones; MG-Hs (MG-H1/H2/H3), methylglyoxal-hydroimidazolones; GOLD, glyoxal lysine dimer; MOLD, methylglyoxal lysine dimer; GALA, N^6^-glycoloyl lysine; GOLA, glyoxal lysine amide; MOLA, methylglyoxal lysine amide; GLA, glyoxal lysine amidine; MGLA, methylglyoxal lysine amidine; LM, *N*^ε^-3-hydroxypropyl-lys; GALA, *N*^6^-glycoloyl lysine; Arg-P, argpyrimidine; Pent, pentosidine; CEA, *N*^7^-carboxyethyl arginine. CMA, *N*^7^-carboxymethyl arginine; CMC, S-(carboxymethyl)cysteine; 2-SC, S-(2-succinyl)cysteine. TCA: trichloroacetic acid; NFPA: nonafluoropentanoic acid; COF: covalent organic frameworks; OPD: o-phenylenediamine; OPA: o-phtalaldehyde; DNS-Cl: dansyl chloride; ECF: ethyl chloroformate; FMOC-Cl, 9-fluorenylmethyl chloroformate; TFA, trifluoroacetic acid anhydride; SPE, solid-phase extraction; SPME, solid-phase microextraction; LTLT, low-temperature long-time; HTST, high-temperature short-time pasteurization; and ESL, ultra-pasteurization; UHT, ultra-high-temperature; BS, in-bottle sterilization; WPH, whey protein hydrolysate; SPH, soy protein hydrolysate; CPH, collagen protein hydrolysate.

**Table 2:** Comparison of different pretreatment processes for determination of free AGEs in foods.

Sample	Sample Pretreatment Processes					AGE	Performance Level * (1 → 5)				Ref.
	Solvent Extraction, Dilution/Filtration	Deproteinization	Defatting	Reduction with NaBH_4_	SPE/SPME		Time Cost	Operability	Accuracy/ Recovery	Economic Cost	
Soy sauce	Dilution					CML	5	5	2	5	[78]
	Dilution	75% MeOH					5	5	2	5	
					SPE		4	4	3	3	
		75% MeOH			SPE		4	4	4	3	
Beer	Membrane filter (0.45 μm)					F-Lys, Pyr, MG-H1, Arg-P, etc.	5	5	2	5	[107]
Sy sauce, Beer, Fish, Sausage, Nuts, Gruel, Infant formula		Sulfosalicylic acid, NFPA			SPE	CML, CEL, Pyr, G-H1, MG-H1, GOLD, MOLD, Arg-P, Pent	4	4	3	3	[85,104,108,120]
Milk		Acetone/MeOH	n-Hexane		SPE	CML, Pyr, F-Lys	3	3	4	3	[103]
Canned fishes, Cheeses, Cookies		MeOH/CHCl_3_	MeOH/CHCl_3_		SPE	CML, CEL	3	3	3	3	[94,95,96]
Meats, Fishes, Roasted patties		TCA, 80% CH_3_CN	n-Hexane		SPE	CML, CEL	3	3	4	3	[92,117,118,121,126]
Cookies		TCA		4 °C, overnight	SPE	CML, CEL	2	4	4	3	[98]
Chicken, fish		TCA	n-Hexane		SPME	CML, CEL, Pyr	3	4	4	3	[127]

* Performance level: Time cost (long 1 → 5 short), Operability (complex 1 → 5 simple), Accuracy/Recovery (low 1 → 5 high), Economic cost (expensive 1 → 5 inexpensive).

**Table 3:** Comparison of different pretreatment processes for determination of bound and total AGEs in foods.

Sample	Sample Pretreatment Processes						AGE	Performance Level * (1 → 5)				Ref.
	Protein Fractionation	Reduction with NaBH_4_	Defatting	Hydrolysis	Derivatization	SPE/SPME		Time Cost	Operability	Accuracy/ Recovery	Economic Cost	
Whey proteins				6 M HCl, 110 °C, 20 h			CML, CEL, MG-H1	2	5	1	5	[122]
UHT milk				Microwave, 6 M HCl, 150 °C, 1 min, 165 °C, 10 min			CML, CEL, G-Hs, MG-Hs, GOLD, MOLD, Arg-P, Pent, etc.	5	5	2	5	[71,75]
Infant formula				6 M HCl, 110 °C, 20–24 h		SPE	CML, CEL	2	4	3	3	[85,87,88]
Manuka honey	Dialysis, Precipitation			6 M HCl, 110 °C, 23 h			CML, CEL, F-Lys, MG-H1,	1	3	2	5	[106]
Beer	Dialysis, 4 °C, 4 day			6 M HCl, 110 °C, 23 h			F-Lys, MG-H1, Arg-P, etc.	1	3	2	5	[107]
Soy sauce, Coffee		4 °C, 24 h or RT, 4 h		6 M HCl, 110 °C, 20 h			CML, CEL	1	3	3	5	[78,82]
Bread crust		20 °C, 4 h		8 M HCl, 110 °C, 23 h			CML, CEL	2	3	2	5	[114]
Milk, infant formula, cereal, nuts and meat products		RT, 4 h		Microwave, 6 M HCl, 150 °C, 1 min, 165 °C, 10 min			CML, CEL, G-Hs, MG-Hs, GOLD Arg-P	4	4	4	5	[130]
Dairy products, Milk, Meat, Roasted beef patties		RT, 4 h or 4 °C, 12 h or overnight		6 M HCl, 110 °C, 20–24 h		SPE	CML, CEL, F-Lys	2	3	4	3	[74,77,103,112,117]
Milk,	Precipitation with TCA	RT, 2 h		6 M HCl, 110 °C, 23 h			CML, CEL, Pent	2	3	3	4	[102]
Milk powder, Soy sauce, Meat and fish	Precipitation with TCA, MeOH/CHCl_3_	4 °C, 8–12 h or overnight		6 M HCl, 110 °C, 24 h		SPE	CML, CEL	1	2	4	3	[78,111,118,126]
Baked and fried foods	Sonication with acetone	RT, 4 h		6 M HCl, 110 °C, 24 h		SPE	CML, CEL	2	2	4	3	[129]
Cookies	Precipitation with TCA			6 M HCl, 110 °C, 24 h		SPE	CML, CEL	3	3	4	3	[98]
Commonly consumed foods, Bread	Precipitation MeOH/CHCl_3_	RT, 4 h 25 °C, 3 h		6 M HCl, 110 °C, 20–24 h			CML, CEL, G-H1, MG-Hs, GOLD, MOLD, Arg-P, Pent, etc.	2	3	4	4	[110,131]
Roasted pork and chicken, UHT milk			CH_2_Cl_2_/ MeOH	Microwave, 6 M HCl, 150 °C, 1 min, 165 °C, 10 min			CML, CEL, G-Hs, MG-Hs, GOLD, MOLD, Arg-P, Pent, etc.	5	5	2	4	[132]
Plant-based burgers		25 °C, 4 h	n-Hexane	6 M HCl, 110 °C, 24 h			CML, CEL	2	4	4	4	[119]
Canned meat and seafood product		RT, 4 h	CH_2_Cl_2_/ n-hexane	6 M HCl, 110 °C, 24 h			CML, CEL, G-H1, MG-H1, GOLD, MOLD, Arg-P	2	4	4	4	[128]
Raw and cooked meat		RT, 4 h	CHCl_3_/ MeOH	6 M HCl, 110 °C, 20 h	OPA		CML	2	2	4	3	[72,73]
Raw pork, roast pork	Precipitation water/MeOH	RT, 1 h	Petroleum ether	6 M HCl, 110 °C, 20 h			CML, CEL, GOLD, MOLD, etc.	2	2	2	4	[109]
Dairy products, Tea, Canned fishes, Cookies, Milk		RT or 20 °C, 4 h, 4 °C, overnight	n-Hexane, CHCl_3_/ MeOH	6–8 M HCl, 110 °C, 24 h		SPE	CML, CEL	1	2	4	2	[94,95,96,97,100,101,115,116]
Chicken and fish products		4 °C, overnight	n-Hexane	6 M HCl, 110 °C, 24 h		SPME	CML, CEL, Pyr	1	3	4	2	[127]
Roasted nuts			n-Hexane/MeOH	6 M HCl, 110 °C, 18 h	SOCl_2_/MeOH TFA		CML	3	3	3	3	[69]
Infant formulas, Fried fish	Water/MeOH/CHCl_3_	4 °C, 6 h or overnight	CHCl_3_/acetone	6 M HCl, 110 °C, 18–24 h	SOCl_2_/MeOH TFA		CML	1	1	3	3	[67,68]
Fish cakes, Fried shrimp	CHCl_3_/acetone	4 °C, 8 h	CHCl_3_/acetone	6 M HCl, 110 °C, 24 h			CML, CEL, MG-H1	2	2	2	3	[124,125]
Coffee, Cereal foods, Dairy products	60% TCA, MeOH/CHCl_3_	4 °C for 6–12 h or overnight	n-Hexane, Petroleum ether	6 M HCl, 110 °C, 24 h or 160 °C, 35 min		SPE	CML, CEL	1	1	4	3	[81,86,100]
Food items	HClO_4_	4 °C, overnight	CHCl_3_/ MeOH	6 M HCl, 110 °C, 20 h	ECF	SPE	CML	1	1	4	2	[83]
Processed foods	TCA	4 °C, overnight	n-Hexane	6 M HCl, 110 °C, 24 h	FMOC-Cl	SPE	CML, CEL	1	1	4	2	[113]
UHT milk, Infant formulas				MED, 37 °C, 24 h × 3			Pyr,	1	4	3	3	[70,71,75]
Dairy products				MED, 37 °C, 24 h × 3		SPE	CML, F-Lys, Pyr,	1	4	4	2	[84,103]
Manuka honey, Beer	Dialysis, 4 °C, 4 days			MED, 37 °C, 24 h × 3			CML, CEL, Pyr, F-Lys, MG-H1, Arg-P, etc.	1	3	3	3	[106,107]
Bread	Precipitation, water/2-ProOH Ultrafiltration			MED, 37 °C, 24 h × 3			CML, CEL, G-Hs, MG-Hs Pyr,, etc.	1	3	3	3	[110]
Bakery products, Almond	Dialysis		n-Hexane, Petroleum ether	MED, 37 °C, 24 h × 3			CML, CEL, Pyr, F-Lys, MG-H1, Arg-P, Pent	1	3	4	2	[104,105]
Beer, Sausage	Lyophilization		n-Hexane	MED, 37 °C, 24 h × 3	DNS-Cl.		CML, CEL, G-Hs, MG-Hs, GOLD, MOLD, Pyr, etc.	1	3	4	2	[76]

* Performance level: Time cost (long 1 → 5 short), Operability (complex 1 → 5 simple), Accuracy/Recovery (low 1 → 5 high), Economic cost (expensive 1 → 5 inexpensive). RT: room temperature; MED: multi enzymatic digestion (pepsin, Pronase E, leucine aminopeptidase, prolidase).

**Table 4:** Comparison of different analytical techniques for determination of AGEs in foods.

Techniques	Exampling AGEs	Advantages	Disadvantages	References
GC-MS	CML, CEL	High sensitivity and selectivity; Easy operation; Fast data acquisition	Time-consuming sample preparation; Requires derivatization of AGEs; Low accuracy and stability; Limited application for AGEs analysis	[67,68,69]
HPLC-UV; HPLC-DAD	UV-sensitive AGEs (pyrraline, etc.) or derivatives	Low-cost; Easy operation; Sensitive to targets	Requires derivatization of UV-inactive AGEs; Limited application; Not be entirely specific to AGEs	[70,71]
HPLC-FLD	Fluorescence AGEs (pentosidine, etc.) or derivatives	Low-cost; Easy operation; Sensitive to targets (superior to HPLC-UV)	Requires derivatization of nonfluorescent AGEs; Limited application; Lacks of specificity	[72,73]
LC-MS UHPLC-MS	Common AGEs	High sensitivity and selectivity; Easy operation; No derivatization	Expensive equipment; Relatively large reagent consumption; Complex sample preparation and sample losses; Highly trained personnel	[74,75,76,77,78,84,101]
LC-MS/MS	Common AGEs	High sensitivity and selectivity (superior to LC-MS); High precision and high resolution; Easy operation; No derivatization	Expensive equipment; Relatively large reagent consumption; Complex sample preparation and sample losses; Highly trained personnel	[79,80,81,82,83,85,86,87,88,89,90,91,92,93,94,95,96,97,98,99,100,102,103,104,105,106,107,108,109,110]
UHPLC-MS/MS; UPLC-MS/MS	Common AGEs	High sensitivity; No derivatization; Reduced solvent consumption; Short analysis time; Good repeatability and reproducibility	Expensive equipment; High maintenance costs; Complex sample preparation; Highly trained personnel	[102,111,112,113,114,115,116,117,118,119,120,121,122,123,124,125,126,127,128]
UHPLC-Orbitrap-Q-Exactive-MS	Common AGEs	High sensitivity; No derivatization; Short analysis time	Expensive equipment; High maintenance costs; Not suitable for quantitative analysis; Highly trained personnel	[129,130,131,132]
ELISA	CML, pentosidine, MG-derivatives	Fast and convenient; Easy operation; High sensitivity and specificity; Wide application	Requires specific antibodies; Cross-reactivity with other compounds; Semi-quantitative; Results cannot be compared with instrumental studies; Susceptible to matrix effects; Requires trained personnel and specialized equipment	[83,85,133,134,135]

**Table 5:** Contents of main AGEs detected in commonly consumed foods.

Food Sample *	Form	Contents (μg g^−1^ or μg mL^−1^) **										References
		Noncross-Linked AGEs				Cross-Linked AGEs			Noncross-Linked AGEs			
								Arg-Derived AGEs				
		Lys-Derived AGEs										
		CML	CEL	F-Lys	Pyr	GOLD	MOLD	Pent	G-Hs	MG-Hs	Arg-P	
*Meat and Meat products*												
Raw meat and fish	B	0.1–68.7										[111]
Meat dishes	B	1.0–424										[111]
Beef meatballs	T	7.27–14.44										[73]
Cooked meats	T	1.07–21.84										[72]
Cooked meats (8)	B	7.19–17.2										[83]
Cooked fish (4)	B	9.81–16.9										[83]
Fried fish nuggets	T	29.26–59.17										[68]
Stewed meat (beef, pork, mutton)	F	0.057–0.340	0.061–0.204									[121]
Roasted beef patties	F B	0.08–0.16 3.41–3.96	0.015–0.021 0.86–1.04									[117]
Processed meat (11)	B	16.0–305	3.84–107									[114]
Raw and sterilized pork	F B	0.27–7.99 1.96–8.75	0.12–1.14 1.18–8.04									[118]
Roasted beef patties	T	74.58	21.09									[115]
Ground beef	B	2.76–19.96	2.32–11.89									[89]
Raw and sterilized beef, pork, chicken	F B	0.21–0.71 2.39–16.56	0.12–7.51 1.16–15.73									[91]
Plant-based burgers	T	35–110	25–110									[119]
Raw and heated fish	F B	0.11–1.29 0.16–5.50	0.10–6.20 2.80–6.03									[92]
Canned fishes	T	0.52–69.24	18.75–243.9									[96]
Raw and heated ground beef	B	2.76–19.96	2.32–11.89									[89]
Sausage	F	1.29–6.58	5.24–16.32									[120]
Fried beef	T	12.86–16.99	8.97–17.73									[135]
Fried chicken	T	10.05–16.57	5.96–15.08									[135]
Fried fish	T	0.63–5.12	17.24–17.61									[135]
Fish (boiled, canned, roast, fried)	F B	0.09–0.13 0.03–0.06	0.07–0.09 3.45–27.18		0.17–23.27 0.02–11.36							[127]
Chicken (boiled, canned, roast, fried)	F B	0.31–0.36 0.74–0.82	0.11–0.15 0.14–37.29		3.35–26.09 0.43–8.72							[127]
Fried shrimp	B	3.61–22.43	75.9–304.2				29.2–341.6					[125]
Cooked meats (47):	B	0.8–48.2	0.4–77.1				2.1–69.5					[123]
Cooked fishes (11):	B	0.5–11.7	0.6–28.2				0.9–109					[123]
Roast pork	B	9.2	40					0.5	0.2			[109]
Beef and pork salami	T	143–175	169–354			Trace	16–17	Trace–29				[130]
Canned pork	T	25.08–35.83	47.20–130.4			4.68–12.94	82.6–323.9	2.24–33.09	2.04–15.77	0.27–0.87		[128]
Canned chicken	T	5.03–35.74	22.58–132.0			3.07–7.38	26.7–140.5	1.03–2.28	2.04–15.77	ND–0.22		[128]
Canned spam	T	12.06–20.66	27.60–79.22			3.91–8.93	53.59–94.0	0.99–2.88	0.71–1.54	ND–0.06		[128]
Canned snail	T	18.87–32.94	29.23–121.5			14.49–24.86	129–285	0.55–4.29	0.75–2.49	0.07–0.55		[128]
Canned saury	T	39.82–60.88	93.28–255.7			6.13–11.32	93.3–191.4	2.61–4.88	0.88–1.79	ND–0.26		[128]
Canned eel	T	21.99–97.76	82.52–189.4			2.27–7.41	65.9–241.4	1.30–16.54	ND–5.26	ND–0.55		[128]
Canned mackerel	T	23.42–80.88	34.34–278.5			5.58–14.52	43.8–300.3	0.49–22.09	ND–7.92	ND–0.94		[128]
Canned tuna	T	9.62–40.65	35.77–129.6			2.76–4.02	19.0–46.8	ND	ND	ND		[128]
Canned sardine	T	13.32–129.9	16.42–135.2			1.06–7.64	15.4–130.2	ND–11.91	ND–6.47	ND		[128]
Canned clam	T	4.25–44.19	11.76–60.39			6.78–22.61	34.0–66.6	ND–5.18	ND–0.63	ND		[128]
Fish cakes	T	0.85–3.14	5.47–13.51			7.89–14.47						[124]
Roasted pork and chicken	T	ND–3.2	<LOQ–37			ND–68	ND–55.9	5.35–15.83	ND–<LOQ	ND–<LOQ	ND	[132]
Beef, pork, chicken, meat products (54)	B	0.42–39.15	0.63–44.18			0.47–27.48	1.32–159.0	0.40–10.15	0.20–13.20	ND	ND–1.11	[131]
Fish and seafood products (20)	B	0.22–23.41	0.20–21.32			ND–10.23	0.18–40.21	ND–2.27	ND–1.42	ND	ND	[131]
***Eggs and Dairy products***												
Raw egg yolk (16)	B	1.50–3.45	1.26–10.57									[90]
Raw egg white (16)	B	0.25–2.01	0.49–5.85									[90]
Cooked egg yolk (16)	B	3.74–7.72	3.19–6.07									[90]
Cooked egg white (16)	B	4.69–9.88	2.39–5.42									[90]
Eggs (3)	B	0.6–4.2	0.7–5.2				0.8–45.0					[123]
Cooked eggs (boiled, pan-fried, omelet) (9)	B	1.51–17.61	0.72–6.32			1.61–63.78	3.09–26.58	0.47–26.69	ND–6.24	ND	ND	[131]
Dairy products	B	ND–17.6										[111]
Sterilized milk	T	1.79–6.05										[111]
Yogurt and cheese	B	2.92–3.40										[83]
Raw milk (4)	B	0.040–0.079										[77]
Processed milk	B	0.028–14.46										[77]
Infant formulas (6)	B	40.0–87.3	0.56–2.34									[113]
Reconstituted milk	T	2.36–3.88	0.35–0.84									[99]
Milk powder and reconstituted milk	T	3–10	0.75–2.75									[116]
Liquid milk (28)	F B	3.5–25 (10^–3^) 35.7–785	0.14–22 (10^–3^) 3.33–836									[100]
Milk powder (26)	F B	0.1–9.0 (10^–3^) 1.9–53.8	0.01–24 (10^–3^) 0.04–128									[100]
Condensed milk (6)	F B	3.0–19 (10^–3^) 28.4–308	0.44–3.5 (10^–3^) 5.71–54									[100]
Milk fats (4)	F B	2.4–8.5 (10^–3^) 134–387	0.38–12 (10^–3^) 31.7–532									[100]
Cheese (9)	F B	0.9–18 (10^–3^) 8.7–442	0.01–2.6 (10^–3^) 0.04–21.8									[100]
Ice cream (6)	F B	0.6–3.0 (10^–3^) 22.3–94	1.12–2.8 (10^–3^) 67.4–123									[100]
Whey protein (3)	F B	6.8–33 (10^–3^) 8.5–46	15–134 (10^–3^) 18.1–206									[100]
Whole milk powder	T	26–154	5–55		0.16–0.58							[101]
Milk Powder	F B	0.36–5.22 2.53–2.56	0.12–1.80 13.78–56.62		ND–0.37 2.85–81.48							[126]
Raw cow milk (4)	F B	3.13–6.02 0.04–0.07		2.68–5.42 1.75–2.29	23.5–55.8 0.16–0.23							[103]
Processed cow milk (6)	F B	2.08–5.49 0.02–14.5		1.30–3.98 1.14–17.2	17.2–214 ND–9.45							[103]
Cheese (11)	B	0.1–3.5	ND–6.3				0.7–27.6					[123]
Milk and milk products (19)	B	ND–22.3	ND–2.6				ND–9.8					[123]
Milk and infant formula	T	14–138	63–98			27–82	Trace	ND–32				[130]
Infant formulas	T	280	180		60	280	190	40	20			[70]
Milk and milk products (28)	B	0.14–14.87	ND–5.30			0.28–7.99	0.09–24.46	ND–3.09	ND–0.33	ND–0.06	ND–0.45	[131]
UHT milk	T	17.94	0.729			1.63–2.49	ND–3.8	0.0328	ND	ND	ND	[132]
***Cereal and Bakery products***												
Bread crust	B	15–140										[114]
Tortilla and potato	B	1.15–2.51										[83]
Cookies	B	6.32–22.84										[77]
Cereals	B	7.6–54.2										[111]
Rice and pasta	B	1.0–4.7										[111]
Sweets & snack	B	6.0–69.6										[111]
Powdered gruel	T	2.3–9.8										[85]
Infant formulas	T	0.5–0.6										[96]
Fried snacks (12)	B	3.91–50.3	0.56–50.5									[113]
Baked snacks (20)	B	2.29–90.3	0.85–57.2									[113]
Protein powder (5)	B	51.0–480	4.07–46.0									[113]
Cookies, biscuit and instant noodles	T	4.07–35.88	1.99–14.49									[129]
Cookies	F B	1.65–2.76 63.74–68.67	1.27–1.99 81.60–103.8									[98]
Cookies (sucrose, butter, egg liquid)	F B	80–220 (10^–3^) 4–6	100–400 (10^–3^) 20–70									[95]
Bread and biscuit	B	14.29–117.5	1.82–71.5									[20]
Potatoes (3)	B	0.1–1.4	ND–0.5				3.0–12.8					[123]
Bread (20)	B	1.3–23.1	0.3–14.4				13.5–231					[123]
Pastry and biscuits (20)	B	0.7–25.9	0.3–34.2				9.7–369					[123]
Cereals and cereal products (15)	B	0.7–19.6	0.2–16.4				13.7–416					[123]
Savory bread spreads (4)	B	13.8–31.1	37.4–68.5				204–445					[123]
Mixed dishes: spring roll, pizza, Russian salad	B	0.6–2.0	0.3–0.7				6.7–15.3					[123]
Bakery Products	B	ND–42	ND–53	Trace–717			ND–218					[105]
Bread (raw dough)	B	2.3	1.6		10.6	2.2	8.5–8.8					[110]
Bread (pre-proofing)	B	2.9	1.7		10.6	2.5	9.3–9.4					[110]
Bread (fermentation)	B	2.4–2.8	1.6–1.9		10.6–10.7	2.4–2.6	0.28–9.7					[110]
Bread (prebaking)	B	3.4–4.9	1.8–2.2		10.6–15.2	2.7–3.0	9.1–11.4					[110]
Bread (baking)	B	6.3	3.1		46	3.7	16–19					[110]
Cookies	B	943	350			3472	2080	60				[130]
Corn flakes	B	152	190			35	1680	Trace				[130]
Salted pretzel sticks	B	272	202			88	1094	39				[130]
Chips	B	67	100			Trace	58	Trace				[130]
Breads (10)	B	2.55–11.58	1.28–10.47			3.23–42.9	8.72–78.7	7.54–21.8	3.44–11.39	ND	1.56–7.51	[131]
Pastry and biscuits (30)	B	1.27–20.89	0.82–10.82			1.41–59.0	3.58–138.6	0.01–14.39	0.01–6.44	ND	ND–4.88	[131]
Sweets and snacks (34)	B	1.53–58.74	ND–16.86			0.44–27.3	3.08–120.0	ND–26.48	ND–4.05	ND–9.22	ND–5.31	[131]
Cereals and cereal products (35)	B	0.84–17.49	0.51–12.64			1.53–31.2	3.50–184.3	0.28–24.83	0.14–7.84	ND	0.20–6.86	[131]
***Fruits, Vegetables and Nuts***												
Fruits and vegetable	B	<LOD–9.5										[111]
Roasted nuts	T	12–17										[104]
Raw almonds (9)	F B	0.298 1.77	0.479 1.92		ND ND							[104]
Roasted almonds	F B	0.520 4.26	0.785 7.70		1.10 12.6							[104]
Fruits (4)	B	ND–1.2	ND–0.1				ND–15.4					[123]
Vegetables (4)	B	0.1–0.7	0.1–0.6				0.1–23.1					[123]
Nuts and seeds (13)	B	2.1–15.8	0.9–33.9				3.2–166					[123]
Roasted nuts	T	126–232	152–211			23–75	219–235	Trace				[130]
Fruits (9)	B	ND–1.21	ND			0.64–4.47	0.10–3.40	ND–0.53	ND–2.01	ND–0.43	ND	[131]
Vegetables (38)	B	ND–2.68	ND–1.84			ND–8.45	ND–20.67	ND–0.99	ND–0.78	ND	ND	[131]
Nuts and seeds (11)	B	3.66–31.56	1.52–42.76			5.92–135.57	7.46–255.6	ND–8.98	ND–0.04	ND–1.76	ND–0.04	[131]
***Beverages***												
Drink mix (27)	T	8.1–131.9										[80]
Commercial coffee substitutes (24) and instant coffees (12)	T	0.17–47										[82]
Coffee beans (roasted)	B	0.008–0.038										[81]
Green teas (44)	T	11.0–96.7	NQ–41.6									[97]
Oolong teas (7)	T	12.9–154	5.6–132.6									[97]
Black teas (41)	T	26.9–309.0	4.6–42.6									[97]
Dark teas (7)	T	95.3–1187.4	13.9–52.6									[97]
WPH-enriched drinks (9)	F B T				ND–77.5 270–761 348–817							[84]
SPH-enriched drinks (9)	F B T				ND–30.5 154–390 162–390							[84]
CPH-enriched drinks (9)	F B T				ND–61.3 226–470 287–469							[84]
Pilsner beer (5)	F B*			11.6–13.6 300–2900	0.16–0.34 132–156		0.49–1.49 47.9–103.0			0.7–1.3 (10^–3^) –		[107]
Dark beer (5)	F B*			8.8–14.9 1800–3200	0.16–0.85 122–400		0.65–1.54 46.2–106.4			0.7–1.9 (10^–3^) –		[107]
Bock beer (5)	F B*			22.2–27.0 1700–2600	0.42–1.63 177–345		1.38–2.47 67.3–90.0			1.4–4.8 (10^–3^) –		[107]
Wheat beer (5)	F B*			9.1–12.5 1000–1400	0.20–0.64 74–203		1.18–2.13 35.9–136.6			0.7–2.0 (10^–3^) –		[107]
Alcohol-free beer (5)	F B*			6.8–12.0 1000–2000	0.16–0.22 55–211		0.31–1.21 35.5–90.4			0.1–1.8 (10^–3^) –		[107]
Alcoholic and non-alcoholic beverages (4)	B	ND	ND–0.1				ND–2.4					[123]
Beer (5)	F	0.07–0.24	0.07–0.14				0.09–2.24					[108]
Beer (3)	T	134.8–147.9	ND–537.1		126.8–177.5	ND	ND–58.6	ND	ND			[76]
***Sauces, Condiments and Other Processed Foods***												
Soups and sauces	B	ND–14.6										[111]
Fresh serrano pepper	B	1.10										[83]
Japanese soy sauce (5)	F B	277–656 1.55–5.27										[78]
Chinese soy sauce (7)	F B	378–988 0.38–2.68										[78]
Soybean products (80)	B	39.1	11.4									[88]
Soy products and vegetarian products (5)	B	0.1–10.0	0.2–7.4				0.5–119					[123]
Sugar, sweets and sweet sauces (16)	B	ND–50.9	ND–20.3				ND–493.3					[123]
Fats, oils and savory sauces (13)	B	ND–12.9	ND–23.5				ND–123					[123]
Tomato soup and pea soup	B	ND–0.4	ND–1.1				ND–22.3					[123]
Soy sauce (7)	F	1.61–9.82	0.57–6.37				ND–7.75	ND–0.19	Trace–0.15			[108]
Sauces and condiment (13)	B	0.11–22.4	0.01–16.50			0.14–16.28	0.33–34.66	0.12–11.95	ND–3.34	ND	ND–1.82	[131]
Soy products (12)	B	0.24–74.42	ND–14.26			0.16–23.61	0.12–39.91	ND–7.92	0.02–14.66	ND–0.08	ND–7.08	[131]
Fats and oils (7)	B	ND–0.51	ND–0.17			ND–1.65	ND–1.15	ND–4.67	ND–1.76	ND	ND–2.25	[131]
Manuka honey	B	0.23–0.55	ND–5.61	0.8–6.9	0.02–0.10		ND–1.38					[106]

* The numbers in parentheses indicate the number of sample species. ** The units of content are μg/g or μg/mL, except for B*, which is μg/g protein. Abbreviations: F, free; B, protein-bound; T, total (free + bound); CML, *N^ε^*-(carboxymethyl)lysine; CEL, *N^ε^*-(carboxyethyl)lysine; F-Lys, fructosyl lysine; Pyr, pyrraline; G-Hs (G-H1/H2/H3), glyoxal-hydroimidazolones; MG-Hs (MG-H1/H2/H3), methylglyoxal-hydroimidazolones; GOLD, glyoxal lysine dimer; MOLD, methylglyoxal lysine dimer; Arg-P, argpyrimidine; Pent, pentosidine; UHT, ultra-high-temperature; WPH, whey protein hydrolysate; SPH, soy protein hydrolysate; CPH, collagen protein hydrolysate.

## Data Availability

No new data were created or analyzed in this study. Data sharing is not applicable to this article.
